# A Genome-Wide, Fine-Scale Map of Natural Pigmentation Variation in *Drosophila melanogaster*

**DOI:** 10.1371/journal.pgen.1003534

**Published:** 2013-06-06

**Authors:** Héloïse Bastide, Andrea Betancourt, Viola Nolte, Raymond Tobler, Petra Stöbe, Andreas Futschik, Christian Schlötterer

**Affiliations:** 1 Institut für Populationsgenetik, Vetmeduni Vienna, Wien, Austria; 2 Department of Statistics, University of Vienna, Wien, Austria; University of Michigan, United States of America

## Abstract

Various approaches can be applied to uncover the genetic basis of natural phenotypic variation, each with their specific strengths and limitations. Here, we use a replicated genome-wide association approach (Pool-GWAS) to fine-scale map genomic regions contributing to natural variation in female abdominal pigmentation in *Drosophila melanogaster*, a trait that is highly variable in natural populations and highly heritable in the laboratory. We examined abdominal pigmentation phenotypes in approximately 8000 female European *D. melanogaster*, isolating 1000 individuals with extreme phenotypes. We then used whole-genome Illumina sequencing to identify single nucleotide polymorphisms (SNPs) segregating in our sample, and tested these for associations with pigmentation by contrasting allele frequencies between replicate pools of light and dark individuals. We identify two small regions near the pigmentation genes *tan* and *bric-à-brac 1*, both corresponding to known *cis*-regulatory regions, which contain SNPs showing significant associations with pigmentation variation. While the Pool-GWAS approach suffers some limitations, its cost advantage facilitates replication and it can be applied to any non-model system with an available reference genome.

## Author Summary

Phenotypic variation is abundant in natural populations, but its genetic basis is not always well-understood. Here, we examine the genetic basis of body pigmentation in *Drosophila*, a trait with a long history of study in *Drosophila* genetics and evolution. We conducted the first genome-wide scan for polymorphism associated with pigmentation variation in a large natural sample of *D. melanogaster*, and found SNPs near two genes, *tan* and *bric-à-brac 1*, affecting the trait. The SNPs associated with pigmentation variation in these genes appear to act by affecting the regulation of the pigmentation genes, rather than their protein coding sequence.

## Introduction

Phenotypic variation is abundant in natural populations, but usually its genetic basis is unknown. That is, even when the biochemical pathways that underlie a trait are well-understood, the identities of the alleles or genes which contribute to variation in natural populations are often not known. This situation, however, is likely to soon change. Statistical genetic approaches originally developed for mapping complex genetic diseases have proved to be successful at mapping phenotypic differences segregating in natural populations, as long as the alleles underlying them are not rare and have reasonably large effects. As a result, these approaches are particularly successful at uncovering the genetic basis of traits that differ due to local adaptation (e.g., [1], [2]) or artificial selection (e.g., [3]–[5]).

Here, we examine the genetic basis of phenotypic variation in *Drosophila* cuticle pigmentation. In genetics, pigmentation has been a classically studied trait— indeed, many of the first markers identified in *Drosophila melanogaster* had pigmentation phenotypes [6] — and has a well-understood genetic basis of moderate complexity, consisting of neither a single Mendelian factor nor hundreds of genes [7]–[10]. For evolutionary genetics, pigmentation has a number of desirable properties. In *D. melanogaster*, pigmentation is highly variable in natural populations due to both genetic polymorphism and phenotypic plasticity [11], [12], can differ between closely-related species [10], [13]–[15], and often does differ between sexes in the *Drosophila* genus [16], [17]. In *D. melanogaster*, in particular, pigmentation shows patterns of spatial variation that suggest that local selection pressures might vary [18]–[20]. The identification of genes responsible for differences in pigmentation can thus shed light on evolution, plasticity and sexual dimorphism in *Drosophila*.

Currently, there are at least 9 genes known to be directly involved in pigment synthesis pathways in *Drosophila* [8], [21], and a number of other genes that can indirectly affect pigmentation patterns through spatial signaling or sex-specific regulation (e.g., *bab* [16] and *Abd-B* [22]). But it is not clear to what extent phenotypic variation in natural populations is due to variation at these loci, or to variation at uncharacterized pigmentation genes. Previously, variation in *D. melanogaster* has been studied *via* either studies of individual genes [18], [19], [23], [24], or by crossing strains with different phenotypes [11], [25]. Although these studies have identified interesting genes and regulatory regions, there has been, to date, no study simultaneously examining the contribution of all genes to pigmentation variation in a large sample of flies, as in a genome-wide association study (GWAS).

To accomplish this, we use a modified GWAS approach, Pool-GWAS, in which individuals with extreme phenotypes are pooled by phenotype, and the pools genotyped and analyzed for differences in allele frequencies (reviewed in [26]). Here, we use pooled next generation sequencing to estimate allele frequency differences between phenotypic classes [27], [28]. As in other GWAS studies, Pool-GWAS uses past recombination events in a large sample of individuals to map phenotypic variation to associated variants genome-wide, and can thus be used to survey much of the natural genetic diversity of an organism. Indeed, the short range over which linkage disequilibrium (LD) decays in *D. melanogaster*—significant LD declines over just 200 bp in most regions of the genome [29]–[31]—makes this species particularly well-suited for identifying the effect of small regions such as short *cis-*regulatory elements controlling pigmentation genes.

## Results/Discussion

**Figure 1:**
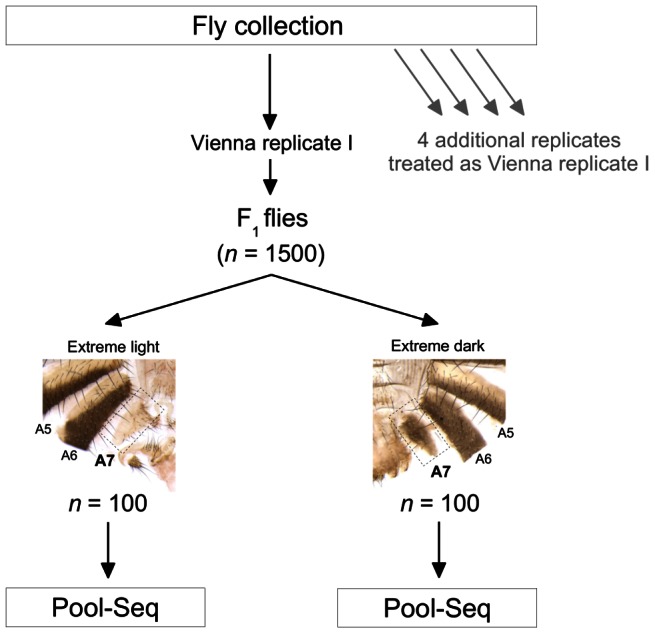
Overview of the experimental design. Wild *D. melanogaster* flies were collected from Vienna, Austria, and Bolzano, Italy, brought into a controlled environment in the laboratory, and treated as shown in the figure. The same procedure was used for all five replicates, with each replicate resulting in 1,500 females for phenotyping, and with 100 light and dark flies from each replicate sequenced.

**Figure 2:**
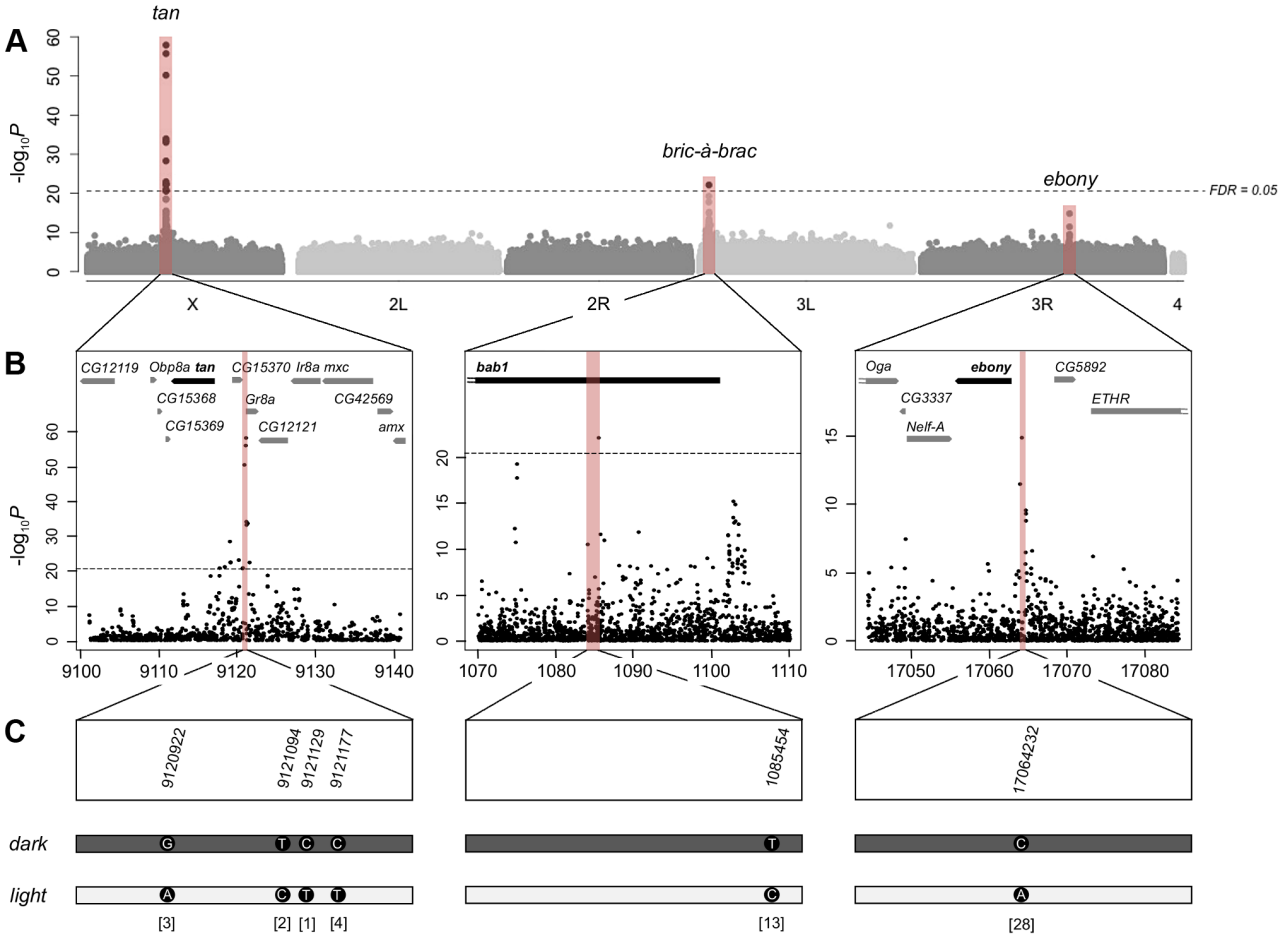
Genome-wide association study of female abdominal pigmentation. A) Manhattan plot for abdominal pigmentation in the full data set (including all five replicates). The–log_10_ *p*-values are plotted against the position on each chromosome. The horizontal dashed line indicates the genome-wide significance threshold at an FDR of 0.05. The red bars indicate candidate genes previously shown to affect pigmentation. B) Detailed view of the *tan* (left), *bab1* (middle) and *ebony* (right) regions. The positions on the x-axis are indicated in kb. The red bars indicate regulatory regions previously shown to affect pigmentation. C) Detailed view of top ranked polymorphisms located within or close to the three pigmentation genes. For every gene, the most significant SNPs fall in regulatory regions.

To obtain material for this study, we collected more than 30,000 wild *D. melanogaster* flies from each of two locations, Bolzano (Italy) and Vienna (Austria). We divided these flies into five independent replicates (two from Vienna and three from Bolzano), cultured a single generation of offspring in a common laboratory environment, and examined the abdominal pigmentation pattern of approximately 8000 of the female offspring (∼1500 females per replicate). We then selected from each replicate 100 of the darkest and lightest flies, as measured by the pigmentation area of the A7 tergite (Figure 1), for whole genome Illumina sequencing. After performing various quality filtering steps, we tested ∼3.3 million SNPs for allele frequency differences between dark and light flies using the Cochran-Mantel-Haenszel (CMH) test, a test designed for meta-analysis of contingency tables summarizing data from different samples. We found 17 SNPs that were significantly associated with female abdominal pigmentation [at a False Discovery Rate (FDR) cutoff of 5%; Text S1]. As expected, these SNPs lie in or near known pigmentation genes, specifically *tan* and *bab1* (Figure 2). Most of the other highly ranked but non-significant SNPs also lie in or near pigmentation genes (79% of the 100 most highly ranked SNPs are within 20 kb from the boundaries of *tan*, *bab1*, or *ebony*; Text S2; Table S1). Interestingly, none of these high ranking SNPs lie in the coding sequence of those three genes, except at *tan*, where there is a single cluster of five synonymous mutations within 60 bp of one another. Instead, the significantly associated SNPs appear to cluster around previously characterized *cis*-regulatory regions of *tan* and *bab1*. For example, in the *tan* region, which harbors all but one of the 17 significant SNPs, a cluster of associated SNPs is located between the upstream genes *CG15370* and *Gr8a*. This location corresponds precisely to the *MSE cis*-regulatory element, which harbors variants responsible for pigmentation differences between the closely-related species *D. santomea* and *D. yakuba* ([15]; Figure 2B, left). Within the limits of correct orthology assignment, none of the putative causal alleles that lighten the cuticle in *D. santomea* correspond to the significant SNPs in this study. In fact, no correspondence would be expected, as *D. santomea* and its close relatives appear to already have the inferred light allele seen here.

Similarly, the most significant SNPs in the *bab* region also correspond to previously described regulatory regions. The *bab* locus consists of two paralogous genes, *bab1* and *bab2*, both affecting abdominal pigmentation, with the segment scored here (A7) affected primarily by *bab1* activity [17], [32]. The significant SNP in *bab1* maps to the “dimorphic” regulatory element in the first intron of this gene, which upregulates *bab1* in females, and represses it in males ([17]; Figure 2B, middle). The remaining highly ranked, but non-significant *bab* SNPs fall into regions that are plausibly also regulatory—a second region within the first intron of *bab1* (downstream of the “dimorphic” element), and a third region near the promoter region of *bab1—*though they have not been validated with functional genetic studies. Finally, the highest ranked SNP outside of the *tan* and *bab* regions lies in the male repressor module of *ebony*, which affects pigmentation in males ([23]; Figure 2B, right), though this SNP was also non-significantly associated with the phenotype, likely due to the conservative criteria used to determine the FDR cutoff (see Text S1).

Thus far, we have considered all replicates from the two populations jointly. To gain insight into the strength and robustness of these associations, we also invested the consistency of the significant SNPs across populations. We therefore analyzed the Viennese and Bolzano samples separately. The *tan* region, which was strongly associated with pigmentation in the joint analysis, remained significantly associated in the separate analyses. All 3 of the SNPs in the Viennese sample that fell below the 5% FDR cutoff, and all 7 in the Bolzano sample, were in the *tan* region (with the three most significant SNPs being identical in the two analyses; Figures S1, S2, S3 and Tables S1, S2, S3). While no significant associations were found with SNPs lying in the *bab* region in the separate analyses, several *bab* SNPs remain highly ranked. We further investigated our power to detect significant associations using only one replicate, i.e., one light and one dark sample from either Vienna or Bolzano (analysis done with 5 paired replicates, not shown). Here, the decline in power is even more apparent; in these analyses, the number of significant associations ranged from 0 to 3 (mean = 0.8 across the 5 replicates at an FDR level of 0.05). This contrast clearly shows the utility of replication, as analyses of single replicates often did not identify even those SNPs with strong effects after accounting for multiple testing.

In general, then, our results are remarkably consistent across populations. This is perhaps not surprising, as European *Drosophila* populations are quite similar genetically [33], and as the CMH test favors associations that are consistent across populations. We were interested, however, in whether or not there were associations specific to either the Vienna or Bolzano populations. Of the four SNPs that were below the 5% FDR cutoff in the Bolzano, but not in the Viennese analysis, only one showed non-overlapping 95% confidence limits for the odds-ratios in the two populations, suggesting that these SNPs were not significant in the Viennese sample due only to a lack of statistical power (note that none of these SNPs were excluded for not meeting the filtering criteria in the Viennese population; data not shown). We further investigated the non-significant associations at *tan*, *bab*, or *ebony* regions for evidence of population-specificity, as population specificity may explain why some SNPs were highly ranked, but non-significant, in the combined analysis. There were some indications of other population-specific associations in the *bab* and *ebony* regions; e.g., SNPs in the *bab1* promoter region discussed above tend to be highly ranked in only one of the populations (Text S3 and Figure S3). One explanation for such differences might be that there are unscored causative variants linked to different SNPs in each population, with two prime suspects for these unscored causal variants being segregating indels and transposable elements. We examined the data for evidence of differences in frequencies of transposable element insertions and indels in the *tan*, *bab* and *ebony* regions. In particular, we looked for indels or TE insertions with larger differences in frequencies than the surrounding SNPs. While some indels in these regions showed suggestive associations with pigmentation, none had frequency differences between light and dark pools that were as large as that of the least significant SNP (which showed an average difference in frequency between light and dark pools of ∼31%; Table S4). In addition, we found no evidence for strong differences in frequency of any transposable element insertions in these regions (using the method in [34]; Table S5).

Thus, we find that female abdominal pigmentation maps to regulatory regions of well-known pigmentation loci. This variation is also associated with sequence variants at *ebony*. Interestingly, regulatory variation in *tan* and *ebony* are also implicated in pigmentation differences between two North American *Drosophila* species (not closely related to *D. melanogaster*; [10]), and *tan* may also play a role in the pigmentation differences found between *D. yakuba* and the island species *D. santomea* [15], [35] (but see [36]). It is tempting to argue that there is some property of *tan* that predisposes it to respond to selection, such as might be the case for the *Melanocortin Receptor 1* gene in vertebrates (reviewed in [37]), though there are other cases where *tan* does not appear to be the main factor affecting pigmentation variation (e.g., [38], [39]). In the case of *D. melanogaster* pigmentation variation, there is some evidence that pigmentation may be under balancing selection [19], [40]–[42], which can elevate causal alleles to intermediate frequencies [43]. Further, balancing selection may have acted on this phenotype long-term, as there appears to be adaptive variation in pigmentation in African *D. melanogaster* [19]; in this case, recombination may have had adequate time to break down linkage disequilibrium between the functional variants and nearby SNPs. As a result, the identification of functional variation mapping to very small regions within the *Drosophila* genome may have been facilitated beyond the genome-wide low linkage disequilibrium in *D. melanogaster* [29]–[31].

As proof of concept, this study demonstrates the power of a Pool-GWAS approach for investigating the genetic basis of natural variation. Comparison to previous studies analyzing the same or similar phenotypes highlights some of the advantages of Pool-GWAS. Our results are consistent with those of [19], who mapped pigmentation variation in a different (though correlated [44]) abdominal segment to the X and 3rd chromosomes in African populations, and were able to associate it to sequence variants at *ebony*. Genome-wide, however, their resolution was limited to the chromosome level. Previous quantitative trait locus (QTL) mapping of female abdominal pigmentation identified only the *bab* region as the major contributor to variation in the trait [11], [25], possibly because the small number of parental strains used in these studies had similar *tan* alleles. Pool-GWAS, however, unlike QTL mapping, surveys a large sample of natural variation, making it less likely that alleles contributing to the trait are excluded from the analysis. A follow-up association study did use a large sample of strains, but focused only on *bab*, and failed to identify individual SNPs strongly associated with pigmentation variation [24]. This result might be due to background variation at the *tan* locus confounding attempts to detect associations at *bab*. Pool-GWAS avoids these pitfalls by simultaneously surveying variation in many strains and genome-wide, revealing that both *tan* and *bab* influence pigmentation.

Pool-GWAS is appealing to biologists working on non-model organisms for various reasons. First, it is low cost relative to many other GWAS approaches—sequencing pools of individuals is far less expensive than individual sequencing [27], and unlike many standard GWAS analyses, it requires no development of SNP chips or resources other than a reference genome. And, as whole-genome sequencing is used, significant associations do not have to rely on high levels of long-range linkage disequilibrium between causal variants and genotyped SNPs; as a result, strong associations can be found with relatively small sample sizes [45]. Finally, while *Drosophila* pigmentation is a well-understood genetic trait, and the associated SNPs map to pigmentation genes, in principle, associated SNPs could have been detected anywhere in the genome, allowing for discovery of loci that affect a trait, but which have not been previously described.

Approaches similar to this one have been attempted in the past. Pooling of samples, of course, has long been a cost saving measure, and has been used in bulk segregant analysis (e.g., [46], [47]), and in standard GWAS (reviewed in [26]), coupled with whole genome sequencing [28], and in a quantitative trait mapping GWAS experiment [48]. Finally, several recent “evolve and resequence” experiments have used pooled sequencing to measure shifts in allele frequencies in populations subjected to multiple generations of natural or artificial selection, in an attempt to discover loci affected by the selection regime (e.g., [49]–[53]). Except for the larger number of generations, these experiments are similar to the process used here. However, in spite of the similarities between this and these previous approaches, there are some important differences. Unlike some of these previous approaches, we use replication as part of the experimental design, which increases the power to detect effects. In contrast to experimental designs requiring crosses or selection sustained over generation, no crosses or laboratory breeding of animals are required (although, for this study, we do rear one generation in a controlled environment). Eliminating this requirement not only reduces labor and costs, but also allows the method to be applied to organisms that cannot be easily bred in the laboratory. In practice, too, the need to propagate from parental populations or strains usually limits the amount of natural variation that can be surveyed, whereas starting with a very large natural sample allows the starting material to capture both large numbers of segregating alleles and as many historical recombination events as possible, rather than including only those that persist during an artificial or natural selection experiment.

Despite offering quite a few advantages, Pool-GWAS also suffers from some limitations. The cost-effectiveness of pooling samples comes at the expense of obtaining reliable estimates of the effects of individual alleles, though it seems that the alleles can be robustly ranked by the strength of their effects (as shown by simulation; Text S2, Figure S4; Table S6 for the ranks of SNPs in *tan* and *bab1*). Like other GWAS methods, Pool-GWAS is expected to have little power to analyze traits with very complex genetic bases, and the power to detect the contribution of rare alleles is limited. Consistent with this, the SNPs with the strongest signals in this study were at high frequencies in the control sample compared to unassociated SNPs [comparison of 100 random SNPs with 17 SNPs with FDR<0.05 : Wilcoxin rank sum test, *p* = 8.1e-05 (Vienna) and *p* = 0.000117 (Bolzano), Figure S10]. To investigate the effectiveness of Pool-GWAS under a range of biological conditions, we performed simulations using a coalescent sample of 8000 haploid individuals, each with a genome roughly equivalent to the euchromatic portion of the *D. melanogaster* genome, and with similar levels of variability and recombination rates (Text S4). These simulations show that, as expected, the power of Pool-GWAS is higher when the minor allele frequencies of causal SNPs are higher, and when there are few causal sites affecting the trait (Figures S5, S6, S7, S8, S9). Pool-GWAS is, therefore, unlikely to be particularly successful for mapping disease loci, which may usually be at low frequencies; rather, we anticipate Pool-GWAS will be best implemented as a means of uncovering the genetic basis of ecologically interesting traits in non-model organisms in a cost-effective manner.

## Methods

### Sample Collection

*D. melanogaster* were collected from vineyard waste heaps, with approximately 30,000 flies collected in Vienna, Austria in October 2010, and a similar number in Bolzano, Italy in September 2011. After collection, flies were randomly divided into two experimental replicates for Vienna or three replicates for Bolzano, put into fresh culture bottles at a density of ∼200 flies per bottle, and allowed to lay eggs at 25°C for 2–4 hours. Bottles were kept at 25°C until the emergence of F1 adults, after which the newly emerged flies were kept at 18°C for several days to allow adult pigmentation to fully develop [12].

### Pigmentation Scoring

We scored approximately 3,500 adult F1 females from Vienna and almost 5,000 adult F1 females from Bolzano for abdominal pigmentation, measured *via* the extent of dark pigmentation on the last tergite (abdominal segment 7, A7) in lateral view. A7 pigmentation reflects overall posterior pigmentation [35], which is more variable than anterior pigmentation in females [12], [54]. To score pigmentation, all flies were initially divided into five pigmentation classes, ranging from 0 (no pigmentation) to 4 (completely pigmented). We then isolated 100 of the lightest class 0 flies and 100 of the darkest class 4 flies from each replicate for sequencing (i.e., a total of 400 and 600 flies from Vienna and Bolzano, respectively). In addition, three sets of control flies (not selected for pigmentation), consisting of between 100–160 flies each, were sequenced for each population.

### DNA Extraction and Sequencing

Genomic DNA was extracted from each pool of female flies by chloroform extraction and ethanol precipitation, and used for paired-end libraries preparation with standard kits and protocols, to obtain fragments of ∼350 bp after gel-purification (or ∼450 bp for all samples from replicate I of the Viennese sample). Libraries were amplified using Phusion DNA polymerase (New England Biolabs, Ipswich, MA) using a 65°C annealing temperature and 10 cycles of amplification, and then purified and quantified using the Qubit HS Assay Kit (Invitrogen, Carlsbad, CA, USA). Libraries were sequenced using the 2×101 bp or 2×151 bp paired-end protocol on either a Genome Analyzer IIx or a HiSeq2000 (see Text S1 for details).

### Mapping of Reads

Following [55], we trimmed raw reads to remove low quality bases and mapped them to the *D. melanogaster* reference genome (v5.18), *Wolbachia pipientis wMel* strain (NC_002978.6) and *phiX174* (NC_001422.1) using BWA [v0.5.8c [56]] with the following parameters: seeding of the reads disabled (−l 200), 1% missing alignments assuming an error rate of 2% (−n 0.01), maximum number of two gap openings (−o 2) and a maximum gap extension of 12 bases (−e 12, −d 12). The mapped reads were filtered for a mapping quality of 20 and for proper pairs with samtools (v0.1.9 [57]).

Due to the overlap in ranges of *D. melanogaster* and *D. simulans* and the difficulty of distinguishing females of these species, we suspected some *D. simulans* contamination among the sequenced flies, which may yield false positive associations as the species can differ in pigmentation [54]. A screen of the wild caught males using diagnostic male genitalia differences [58] yielded an estimate of *D. simulans* contamination of around 1% in both collections. We thus filtered the mapped reads for *D. simulans* contamination, by simultaneously remapping the above reads to five *D. melanogaster* genomes (including the reference genome v.5.18 cited above and those of the MW6, MW28, RAL360, RAL732 strains from the *Drosophila* Population Genomics Project (http://www.dpgp.org/) and five *D. simulans* genomes (unpublished data), using GSNAP version 2011-12-28 [59], [60]. Reads were identified as *D. melanogaster* if they mapped at least as well to *D. melanogaster* as to *D. simulans*, as measured by mapping quality. The effectiveness of this filtering procedure was assessed by inspecting the filtered *D. melanogaster* specific reads for a target set of *D. simulans* specific variants (Text S1 and Figure S11A and S11B); we considered the filtering procedure to be effective when the median frequency of the target set of *D. simulans* alleles was reduced to zero. The filtered reads were then converted to mpileup format using samtools (without quality score adjustment, using option -B), with one replicate-phenotype combination per column.

All further analyses were performed using scripts contained in PoPoolation2 revision 98 [61]. We prepared the mpileup file for each population by converting them to synchronized files, requiring a base quality of at least 20, and by masking indels and repetitive regions (including five flanking nucleotides on both sides of an indel), separately for the two populations. Repetitive regions (e.g. transposable elements, microsatellites) were identified using RepeatMasker v. 3.2.8 (www.repeatmasker.org) with crossmatch version 0.990329 (http://www.phrap.org/phredphrapconsed.html) as the search engine; simple repeats were not masked (Repeatmasker option -nolow). After undergoing all filtering procedures, an average of 112-fold coverage per replicate was obtained (see Text S1 for individual coverage estimates for each of the 16 separate data sets).

### Association Mapping

We tested SNPs showing an association with pigmentation using the Cochran-Mantel-Haenszel (CMH) test, a meta-analysis method for repeated measures of independence, as implemented in PoPoolation2 revision 176 [61]. Essentially, a 2×2 contingency table is created for each replicate, with phenotype (light *vs.* dark) and the two major allele variants at each SNP as the independent nominal variables, and the counts of each allele in each phenotypic category as dependent variables. The CMH test tests independence of the nominal variables across replicates. We analyzed only SNPs that met the following requirements: *(i)* the SNP must contain two alleles that occur at least five times each across all samples, *(ii)* the site must have a coverage of at least 10 in each sample, and *(iii)* to avoid including differences between paralogs and other artifacts due to copy number variation, the site must have a coverage lower than a maximum cutoff set independently for each sample. This maximum cutoff excludes sites with coverage in the upper 2% tail of coverage for all sites. To correct for multiple tests, we used a FDR control (see Text S1 and Figure S12). Indels and transposable element insertions in the regions surrounding the *tan*, *bab*, and *ebony* genes were identified and tested for associations as described in Text S1.

### Feature Analysis of the Best Ranked SNPs

We used SnpEff v2.0.3 [62] and the *D. melanogaster* annotation v5.40 to assign candidate SNPs to genomic features. SNPs not further than 200 bases from the 5′or 3′UTR of a gene were considered upstream or downstream, SNPs further than were considered intergenic. Overlapping or alternative spliced genes were treated separately.

### Accession Numbers

The FASTQ files are available from the European Sequence Read Archive (Accession no. ERP001827; http://www.ebi.ac.uk/ena/data/view/ERP001827).

## Supporting Information

Figure S1 **Manhattan plot for abdominal pigmentation in the Vienna sample.** The–log_10_ *p*-values are plotted against the position on each chromosome. The horizontal dashed line indicates the genome-wide significance threshold at an FDR of 0.05. The red bars indicate candidate genes previously shown to affect pigmentation.
(PDF)

Figure S2 Manhattan plot for abdominal pigmentation in the Bolzano sample. The –log_10_P-values are plotted against the position on each chromosome. The horizontal dashed line indicates the genome-wide significance threshold at an FDR of 0.05. The red bars indicate candidate genes previously shown to affect pigmentation.
(PDF)

Figure S3 Pigmentation loci with differences between Bolzano and Viennese populations highlighted. Shown are SNPs in the *tan* (left), *bab* (middle), and *ebony* (right) regions. The −log *p*-values in this plot were obtained from the joint analysis, combining data from both populations, and not from the analysis of the two populations separately. The horizontal dashed lines indicate the genome-wide significance threshold at an FDR level of 0.05 for the combined analysis. Results from the separate analysis of the populations are shown by the color of the SNPs. SNPs that are highly ranked in both analyses are highlighted in green, SNPs highly ranked only in Bolzano are shown in blue, and SNPs highly ranked only in Vienna are shown in orange.
(PDF)

Figure S4 Comparison of effect estimates from the CMH test and logistic regression on simulated SNPs. Shown are the results of 500 simulations of selection for extreme phenotypes for a quantitative trait. For each simulation, individual and pooled genotypes were collected for 20 SNPs, each with a frequency between 0.4 and 0.6 and a positive or negative effect on the trait mean, drawn from a uniform distribution (with the absolute value constrained between 0.5 and 1 in order to keep the range of estimated effect similar to those of the experiment). An environmental contribution to the trait was simulated by drawing a random normal deviate with a variance equal to that of the genetic variance in the trait. The plotted effect estimates are, for the top panel, the absolute value of the log of the pooled odds ratio estimate from the CMH test, and for the bottom panel, the absolute value of the logistic regression coefficient (already on a log-scale). The magnitude of this effect is a function of the probability of an allele at that SNP causing the individual carrying it to fall into the light or dark extreme. For comparison, the absolute value of the effect on the trait mean is shown on each plot in blue. Note that this is a different quantity than the estimated effects from the statistical tests, so the magnitudes of the two quantities are uninformative, and these are plotted only to show which SNPs have very similar or very different effects on the trait means.
(PDF)

Figure S5 Receiver operator curves (ROC) for simulations with 10 causal loci. The true and false positive rates are shown on the y- and x- axes, respectively, for the case in which there are 10 causal loci and different genetic penetrance (g), with 25% (top row), 50% (middle row) of 100% of the phenotypic value of an individual due to its genotype. Cases where alleles have uniform effect are denoted with Eff = U, and where alleles have exponentially distributed effects with Eff = E. Causal SNPs were either drawn randomly (mf = 0), or required to have a minor allele frequency of 0.2 (mf = 0.2). The black lines show the cases where extreme phenotypes were selected, and the grey lines show where one extreme phenotype was compared to a random sample (as in a case-control experiment). The solid lines show the same results, except that here regions of zero recombination were excluded from the analysis. Each curve was estimated from 50 simulations. See Text S4 for detailed simulation methods.
(PDF)

Figure S6 Receiver operator curves (ROC) for simulations with 20 causal loci. Plots are as before, but summarize simulations in which there were 20 causal loci.
(PDF)

Figure S7 Receiver operator curves (ROC) for simulations with 100 causal loci. Plots are as before, but summarize simulations in which there were 100 causal loci.
(PDF)

Figure S8 Replicability of *p*-values causal SNPs with equal effects. To investigate the extent to which we expect our results to be repeatable given the same causal loci, effects and allele frequencies in the population before selection is applied, we performed some additional simulations. To this end, we used a similar approach as above, but fixed a random seed such that the causal loci and their effects were identical among replicates. Some stochastic effects remain: the division of flies into replicates and change in individual phenotypes due to the environment varied between simulations (the proportion of the phenotype due to the environment was set to 0.5). We then ran 20 simulations and assessed the consistency of results between runs. Different numbers of causal loci were assigned to regions of the simulated genomes corresponding to the *tan* and *bab* regions, with *L* = 1, 5, 10, or 20 per locus. As the strongest candidates from the analysis of the data are at intermediate frequencies in the unselected reference populations, we required the causal loci to have a MAF between 0.2 and 0.5. As an indication of repeatability of the simulation results, we use the range of *p*-values from the CMH test (the maximum of the 20 *p*-values minus the minimum). The range of *p*-values is plotted against the minor allele frequency (MAF) of the causal SNPs. Causal SNPs are shown in the plots as points, with SNPs with high minimum *p*-values shown in gray (*p*-value always >1 e-05, roughly indicating repeatably non-significant SNPs), those with intermediate minimum *p*-values shown in red (minimum *p*-value<1 e-05 and >1 e-07). Note that with loci having equal effects, a higher number of causal SNPs necessarily results in smaller phenotypic effect per locus. The plots show that those simulations with few loci have highly repeatable results, while the effect of the MAF in this restricted range is not very pronounced.
(PDF)

Figure S9 Replicability of *p*-values causal SNPs with exponential effects. Simulations were done as described in the legend for Figure S8, except that the phenotypic effects of alleles were drawn from an exponential distribution. The top plot shows the effect of the MAF, as in Figure S8, while the bottom shows the influence of the phenotypic effects of the alleles.
(PDF)

Figure S10 Allele frequencies in the control samples from Vienna and Bolzano. Shown are frequencies of minor allele frequencies in the control samples from both populations for random vs. highly ranked SNPs. Random SNPs are a randomly drawn subset of the ∼3.3 million SNPs that met our filtering criteria in the combined analysis of the Bolzano and Vienna populations, but which did not occur among the top 200 SNPs ranked by *p*-value.
(PDF)

Figure S11 Result of treatment for *D. simulans* contamination. Samples were treated to filter *D. simulans* contamination as described in the Methods, and then assessed for contamination both before and after treatment. (A) Level of contamination in the four Viennese samples [light and dark samples from both Replicate I (RI) and Replicate II (RII)] before (upper panel) and after (lower panel) treatment. The light sample of Replicate I shows the highest level of contamination of the four samples. (B) Level of contamination in the six Bolzano samples [light and dark from RI, RII, and RIII] before (upper part) and after (lower part) treatment. While the dark samples of the three replicates show a minimal level of contamination, all three light samples are highly contaminated. Note that the contamination was successfully filtered from each contaminated sample in Vienna and Bolzano.
(PDF)

Figure S12 QQ-plot for combined analysis. Quantile-quantile plot for observed *p*-values from the joint analysis and the simulated distribution obtained under the null (obtained using an alpha value of 20; see text for details). The plot shows the–log_10_ *p*-values.
(PNG)

Table S1 Characteristics of highly ranked SNPs in the joint analysis of the Viennese and Bolzano samples. For the top 100 ranked SNPs, the table shows the rank, the location (chromosome and position), the reference and alternative nucleotide, the gene(s) in which they lie (gene ID and gene name), the effect on the gene (synonymous coding, intron, etc.), the change in amino acid and codon (if in coding sequence) and the *p*-value from our analysis. The colors indicate which, if any, pigmentation gene the SNP is near (blue: *tan*; red: *bab1*; yellow: *ebony*; no color: none).
(PDF)

Table S2 Characteristics of highly ranked SNPs in the analysis of the Viennese sample analyzed alone. Values given are as in Table S1, except that the ranking corresponds to the ranking in the Viennese sample.
(PDF)

Table S3 **Characteristics of highly ranked SNPs in the analysis of the Bolzano sample analyzed alone.** Values given are as in Table S1, except that the ranking corresponds to the ranking in the Bolzano sample.
(PDF)

Table S4 **Differences in indel frequencies in the regions near the** *tan*, *bab1* and *ebony* loci. These regions are defined, as before, as 20 kb up- and down- stream of the center of the coding sequence of each gene (within 20 kb of the middle of the coding sequence of these genes); indels are found as described in Text S1 with Dindel. As Dindel divides the reads in these regions into windows, some indels occur in more than one window. In these cases, the presence and absence counts of these indels were usually quite similar, and so we give the average coverage and frequency and the minimum and maximum *p*-values. Similarly, indels with adjacent positions are also merged into a single entry, and the same information is reported for these.
(PDF)

Table S5 Transposable element insertion frequencies in the pigmentation genes *tan*, *bab1* and *ebony*. These regions are defined, as before, as 20 kb up- and down- stream of the center of the coding sequence of each gene. Transposable element insertions found in these regions using the method described in Text S1, except that insertions detected in only one replicate are not shown. A CMH test was performed on the presence and absence counts for each detected insertion. Some frequency estimates may be unreliable, as they come from insertions that overlap with other insertions or, in one case, from an INE-1 insertion, which are typically very short; these are nevertheless shown for completeness.
(PDF)

Table S6 Estimated effects of SNPs with FDR<0.05. The effect of each SNP is ranked by its log odds ratio, as discussed in the text. The *p*-values and odds ratios come from the CMH test used for the main analysis, and ranks are based on the magnitude of the log of the odds ratio. We use the magnitude of the log of the odds ratio to estimate the rank of the effect, as is standard, for the following reason: For a SNP of no effect, the odds ratio should be 1 (and the log odds ratio equal to 0). The size of the estimated effect increases as the odds ratio deviates in either direction from 1 (and the log odds from 0). But, as odds ratios are restricted between 0 and infinity, this deviation is a non-linear function of the size of the real effect. The log odds, in contrast, is symmetric about 0, and the size of this deviation in either direction is expected to be proportional to the size of the effect. Though the effects here are systematically overestimated, the rankings appear to be robust as discussed in Text S2.
(PDF)

Text S1 Supplementary methods and results. Additional detail on the sequencing method, removal of *D. simulans* contamination, statistical analysis for association mapping and FDR correction. Also includes detailed methods on the indel and TE insertion analysis and results.
(PDF)

Text S2 Description of highest ranked SNPs. Describes the characteristics, allele frequencies before selection, and the estimation of effects for the highly ranked SNPs.
(PDF)

Text S3 Results of separate analysis of Bolzano and Vienna populations. Describes additional results obtained for association mapping of pigmentation SNPs done separately for the Bolzano and Vienna samples.
(PDF)

Text S4 Simulation methods. Describes detailed methods for the simulations for examining the effects of different initial allele frequencies, and different genetic architectures of the trait (including the number of loci involved, the distribution of their effects, and different contributions of the environment to the trait).
(PDF)
