# Supplementary figures and images for: A Genome-Wide, Fine-Scale Map of Natural Pigmentation Variation in Drosophila melanogaster
Source: PLoS Genet. 2013 Jun 6;9(6):e1003534. doi: 10.1371/journal.pgen.1003534 (PMC3674992; doi:10.1371/journal.pgen.1003534)

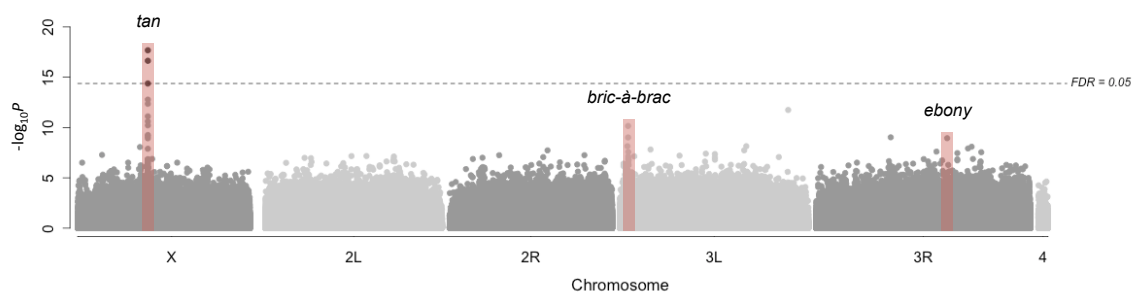

Supplement: Figure S1 — Manhattan plot for abdominal pigmentation in the Vienna sample. The–log10 p-values are plotted against the position on each chromosome. The horizontal dashed line indicates the genome-wide significance threshold at an FDR of 0.05. The red bars indicate candidate genes previously shown to affect pigmentation. (PDF) [file pgen.1003534.s001.pdf]

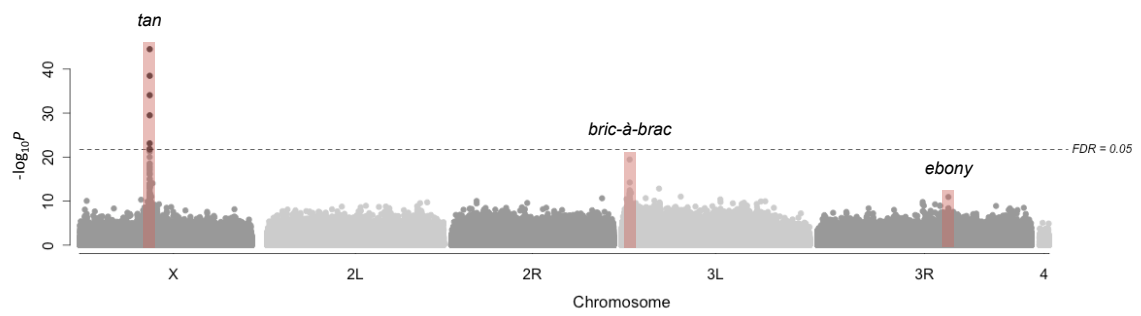

Supplement: Figure S2 — Manhattan plot for abdominal pigmentation in the Bolzano sample. The –log10P-values are plotted against the position on each chromosome. The horizontal dashed line indicates the genome-wide significance threshold at an FDR of 0.05. The red bars indicate candidate genes previously shown to affect pigmentation. (PDF) [file pgen.1003534.s002.pdf]

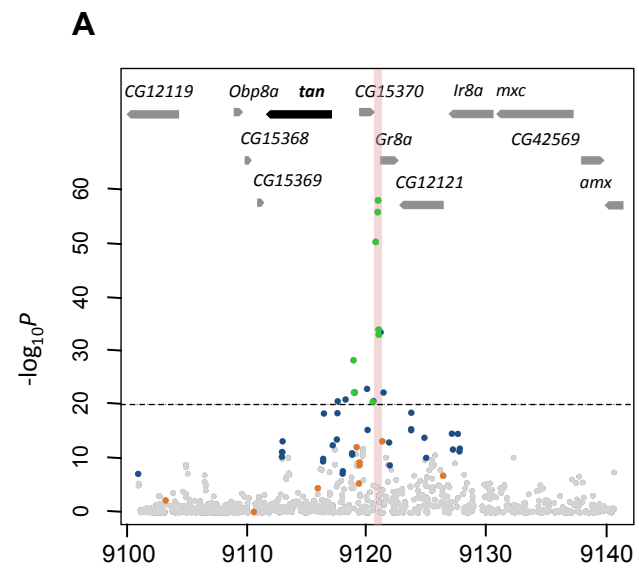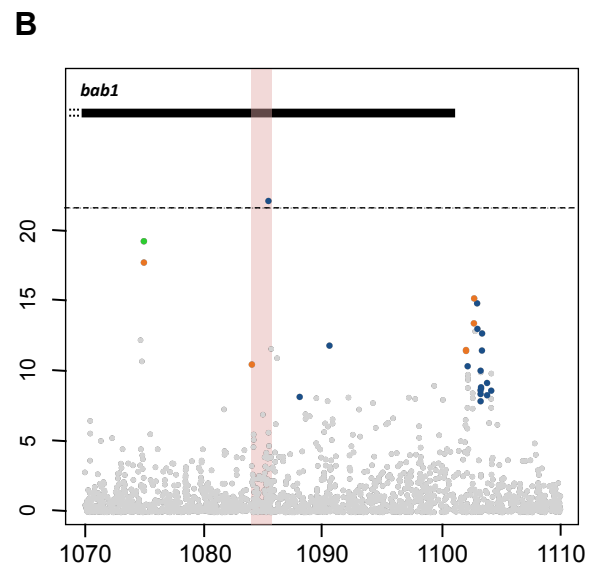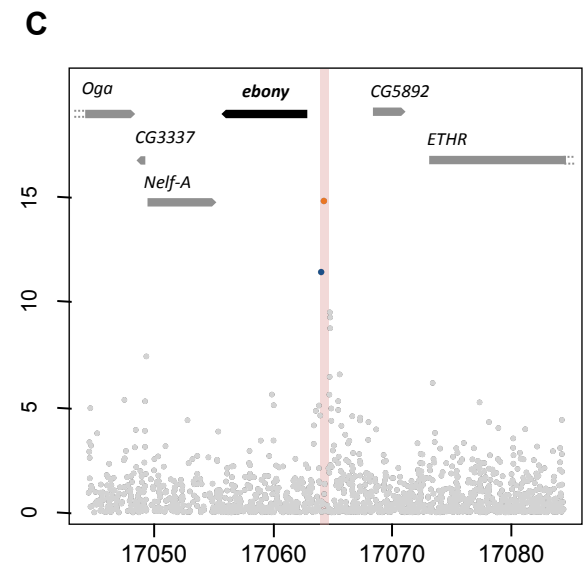

Supplement: Figure S3 — Pigmentation loci with differences between Bolzano and Viennese populations highlighted. Shown are SNPs in the tan (left), bab (middle), and ebony (right) regions. The −log p-values in this plot were obtained from the joint analysis, combining data from both populations, and not from the analysis of the two populations separately. The horizontal dashed lines indicate the genome-wide significance threshold at an FDR level of 0.05 for the combined analysis. Results from the separate analysis of the populations are shown by the color of the SNPs. SNPs that are highly ranked in both analyses are highlighted in green, SNPs highly ranked only in Bolzano are shown in blue, and SNPs highly ranked only in Vienna are shown in orange. (PDF) [file pgen.1003534.s003.pdf]

### CMH test

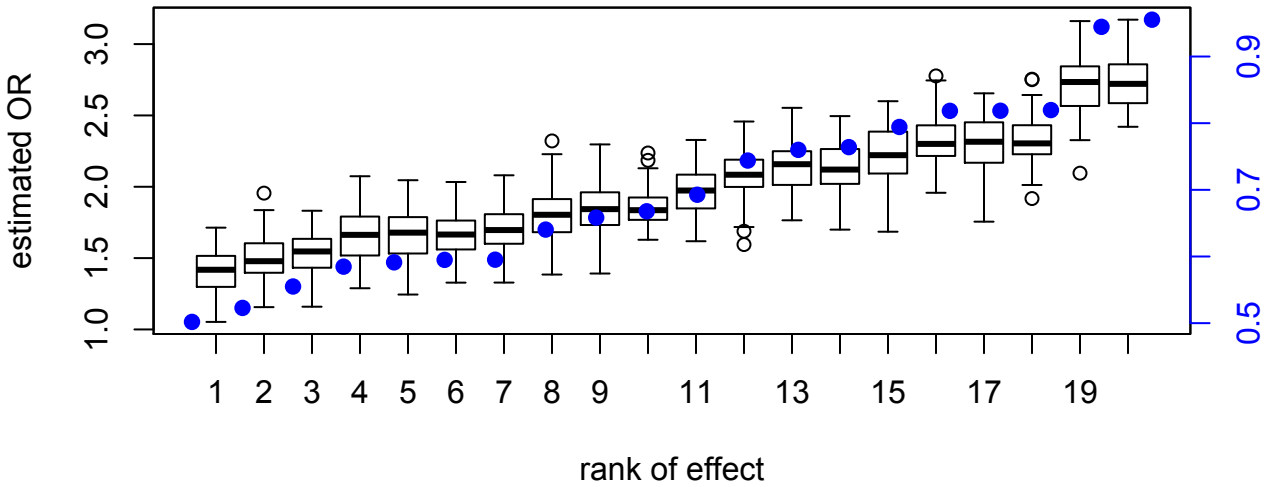

### logistic regression

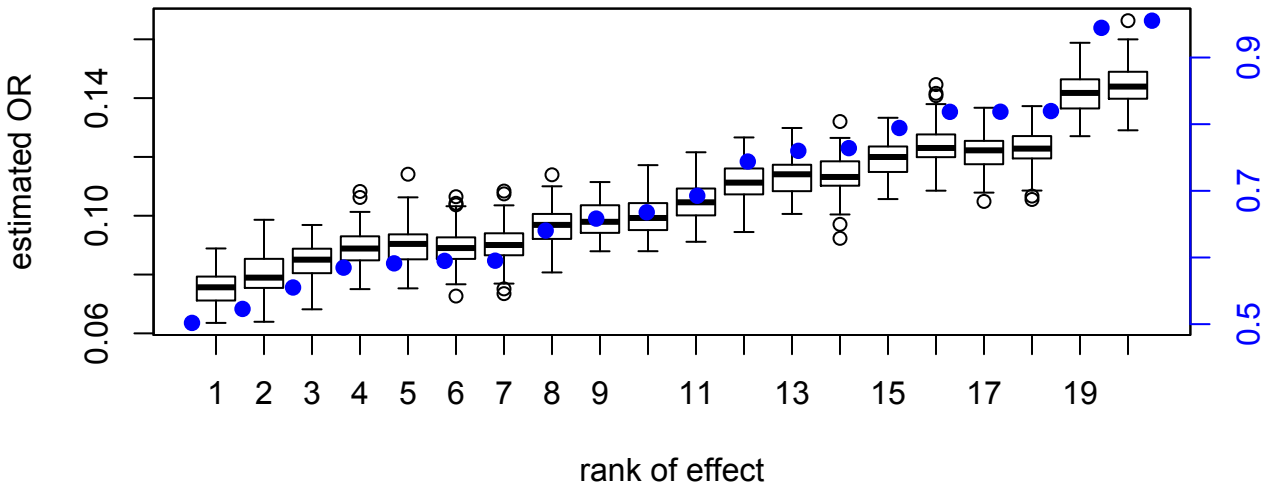

Supplement: Figure S4 — Comparison of effect estimates from the CMH test and logistic regression on simulated SNPs. Shown are the results of 500 simulations of selection for extreme phenotypes for a quantitative trait. For each simulation, individual and pooled genotypes were collected for 20 SNPs, each with a frequency between 0.4 and 0.6 and a positive or negative effect on the trait mean, drawn from a uniform distribution (with the absolute value constrained between 0.5 and 1 in order to keep the range of estimated effect similar to those of the experiment). An environmental contribution to the trait was simulated by drawing a random normal deviate with a variance equal to that of the genetic variance in the trait. The plotted effect estimates are, for the top panel, the absolute value of the log of the pooled odds ratio estimate from the CMH test, and for the bottom panel, the absolute value of the logistic regression coefficient (already on a log-scale). The magnitude of this effect is a function of the probability of an allele at that SNP causing the individual carrying it to fall into the light or dark extreme. For comparison, the absolute value of the effect on the trait mean is shown on each plot in blue. Note that this is a different quantity than the estimated effects from the statistical tests, so the magnitudes of the two quantities are uninformative, and these are plotted only to show which SNPs have very similar or very different effects on the trait means. (PDF) [file pgen.1003534.s004.pdf]

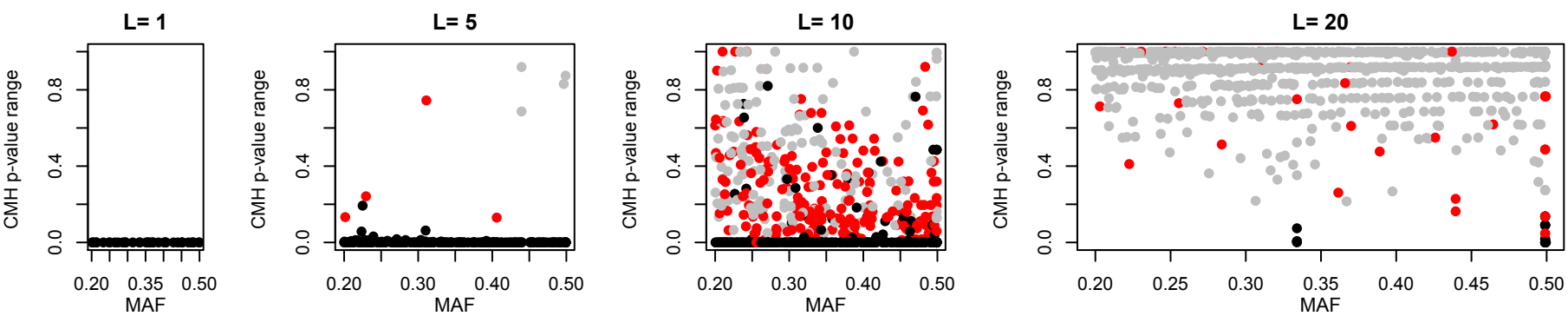

Supplement: Figure S8 — Replicability of p-values causal SNPs with equal effects. To investigate the extent to which we expect our results to be repeatable given the same causal loci, effects and allele frequencies in the population before selection is applied, we performed some additional simulations. To this end, we used a similar approach as above, but fixed a random seed such that the causal loci and their effects were identical among replicates. Some stochastic effects remain: the division of flies into replicates and change in individual phenotypes due to the environment varied between simulations (the proportion of the phenotype due to the environment was set to 0.5). We then ran 20 simulations and assessed the consistency of results between runs. Different numbers of causal loci were assigned to regions of the simulated genomes corresponding to the tan and bab regions, with L = 1, 5, 10, or 20 per locus. As the strongest candidates from the analysis of the data are at intermediate frequencies in the unselected reference populations, we required the causal loci to have a MAF between 0.2 and 0.5. As an indication of repeatability of the simulation results, we use the range of p-values from the CMH test (the maximum of the 20 p-values minus the minimum). The range of p-values is plotted against the minor allele frequency (MAF) of the causal SNPs. Causal SNPs are shown in the plots as points, with SNPs with high minimum p-values shown in gray (p-value always >1 e-05, roughly indicating repeatably non-significant SNPs), those with intermediate minimum p-values shown in red (minimum p-value<1 e-05 and >1 e-07). Note that with loci having equal effects, a higher number of causal SNPs necessarily results in smaller phenotypic effect per locus. The plots show that those simulations with few loci have highly repeatable results, while the effect of the MAF in this restricted range is not very pronounced. (PDF) [file pgen.1003534.s008.pdf]

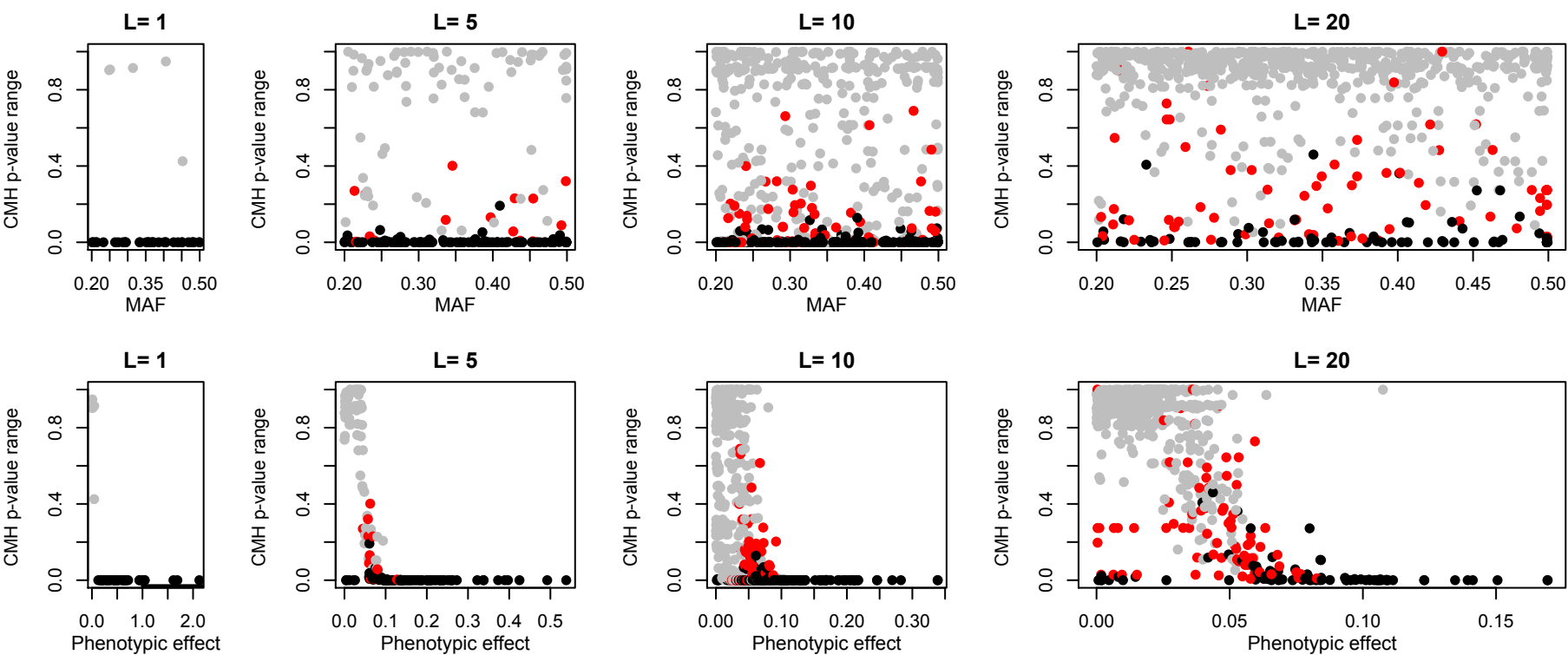

Supplement: Figure S9 — Replicability of p-values causal SNPs with exponential effects. Simulations were done as described in the legend for Figure S8, except that the phenotypic effects of alleles were drawn from an exponential distribution. The top plot shows the effect of the MAF, as in Figure S8, while the bottom shows the influence of the phenotypic effects of the alleles. (PDF) [file pgen.1003534.s009.pdf]

MAF in Vienna control population

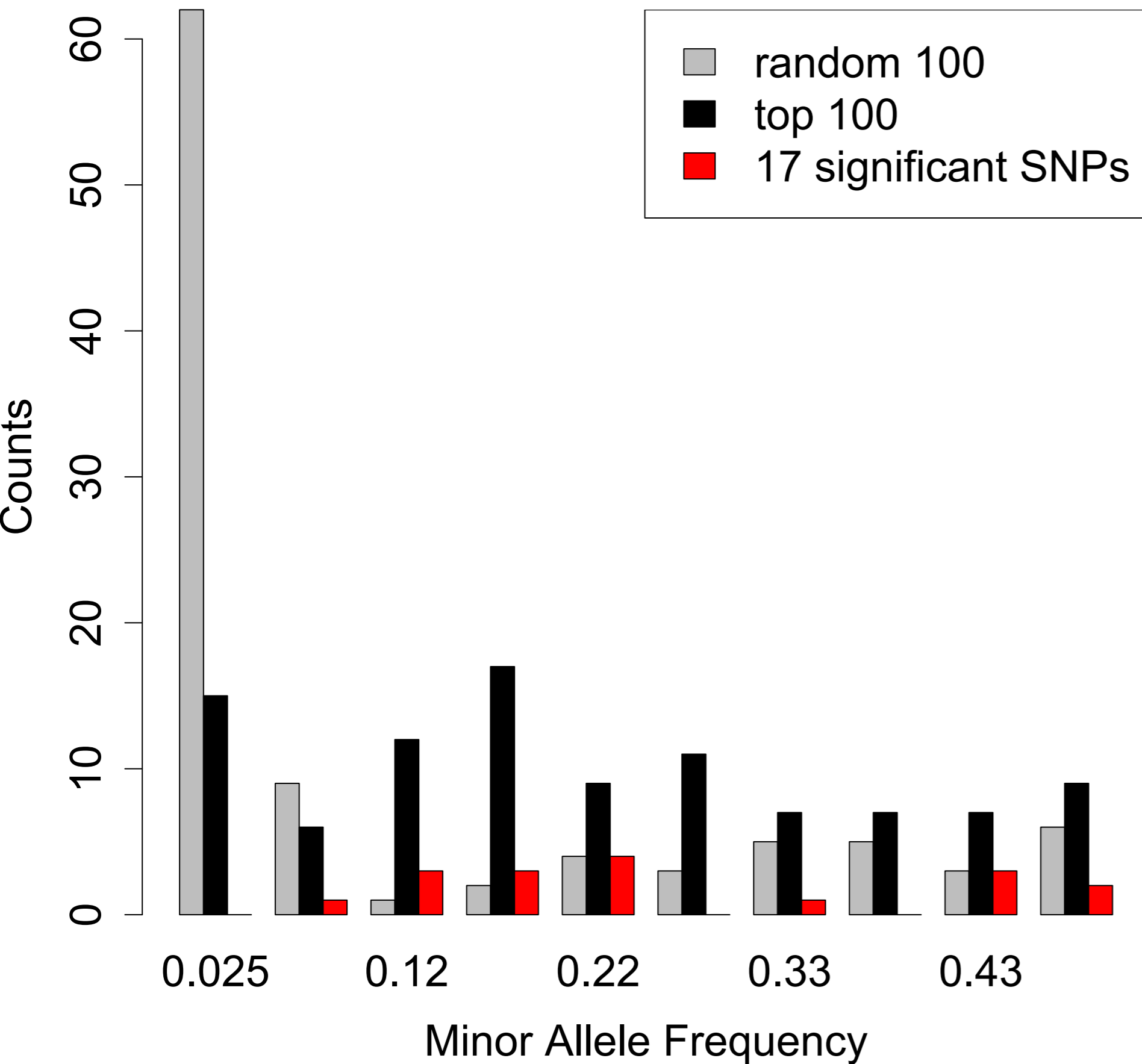

MAF in Bolzano control population

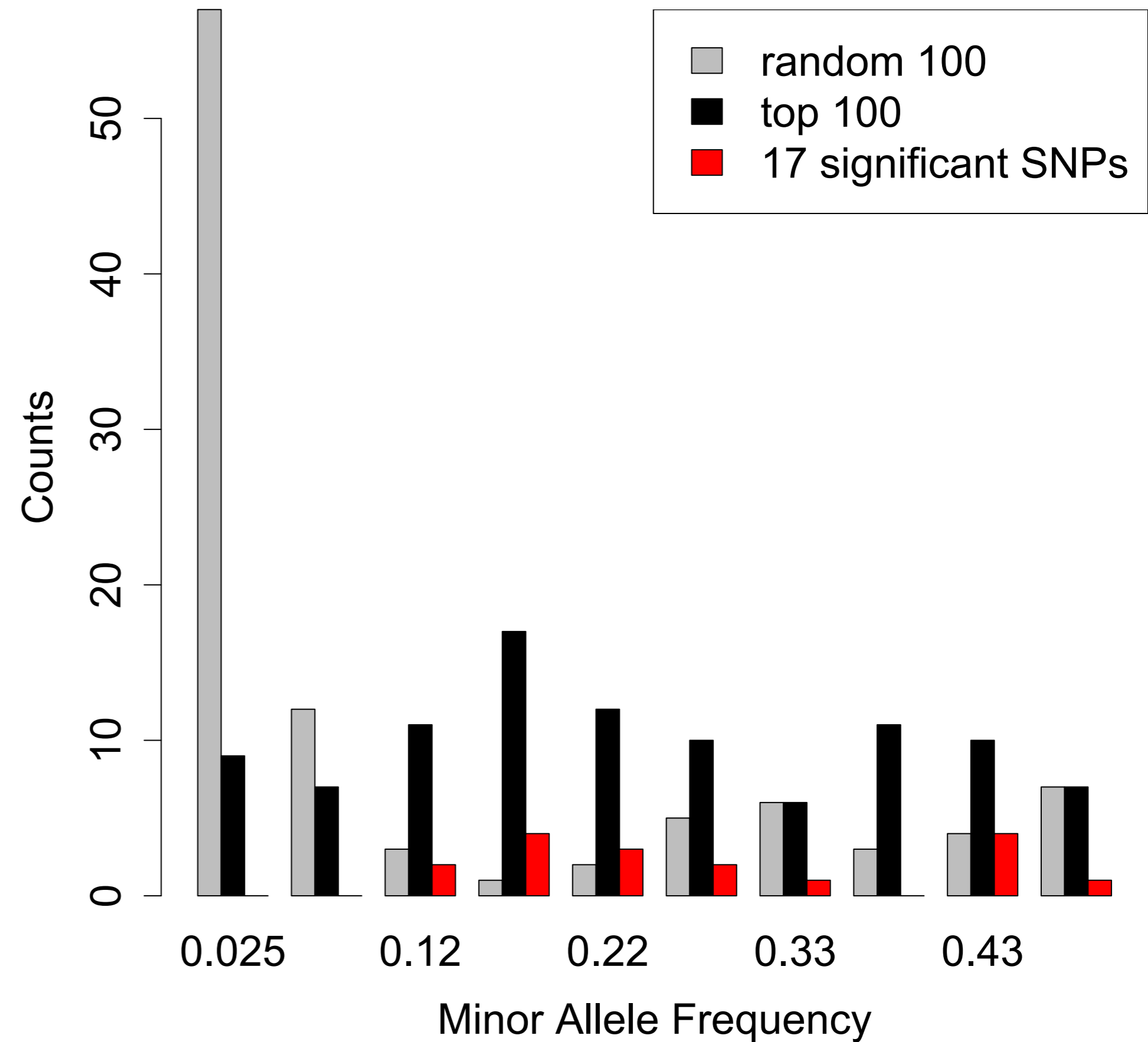

Supplement: Figure S10 — Allele frequencies in the control samples from Vienna and Bolzano. Shown are frequencies of minor allele frequencies in the control samples from both populations for random vs. highly ranked SNPs. Random SNPs are a randomly drawn subset of the ∼3.3 million SNPs that met our filtering criteria in the combined analysis of the Bolzano and Vienna populations, but which did not occur among the top 200 SNPs ranked by p-value. (PDF) [file pgen.1003534.s010.pdf]

**A**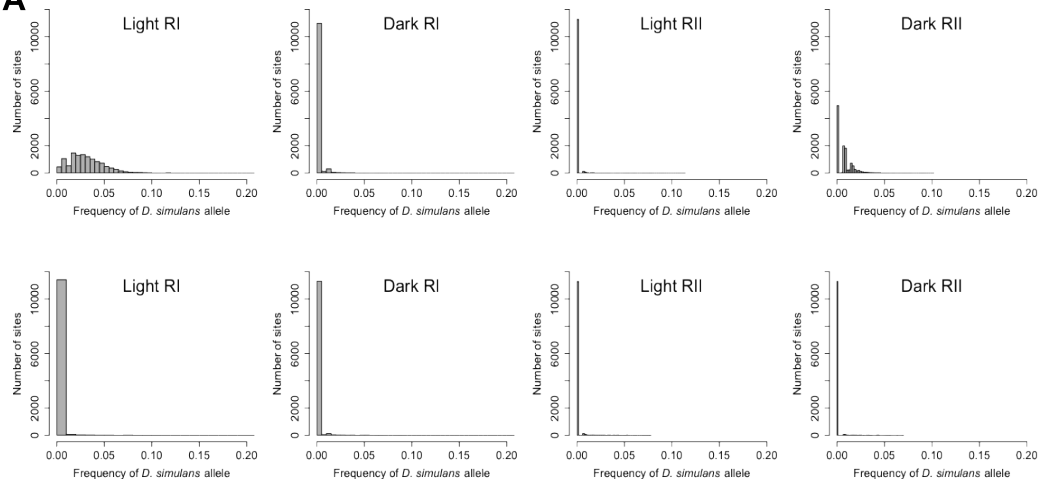**B**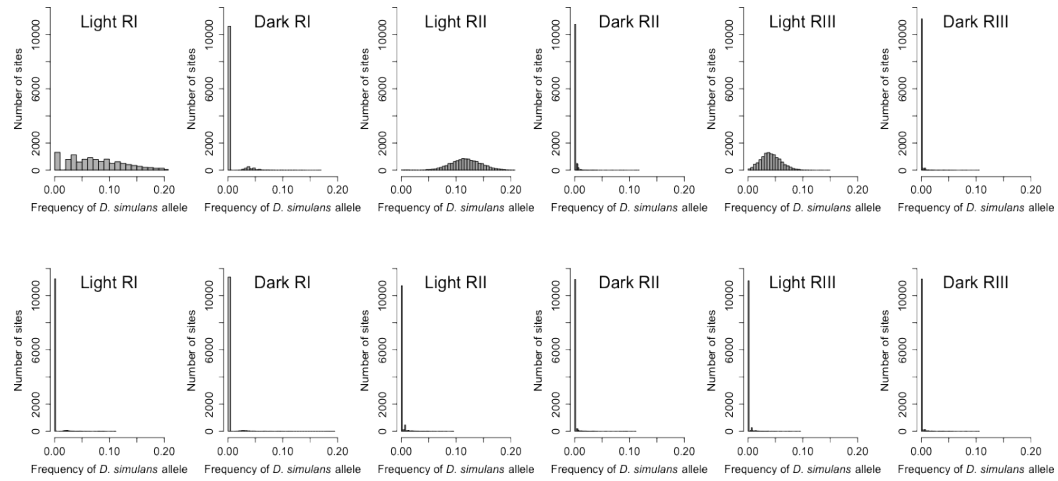

Supplement: Figure S11 — Result of treatment for D. simulans contamination. Samples were treated to filter D. simulans contamination as described in the Methods, and then assessed for contamination both before and after treatment. (A) Level of contamination in the four Viennese samples [light and dark samples from both Replicate I (RI) and Replicate II (RII)] before (upper panel) and after (lower panel) treatment. The light sample of Replicate I shows the highest level of contamination of the four samples. (B) Level of contamination in the six Bolzano samples [light and dark from RI, RII, and RIII] before (upper part) and after (lower part) treatment. While the dark samples of the three replicates show a minimal level of contamination, all three light samples are highly contaminated. Note that the contamination was successfully filtered from each contaminated sample in Vienna and Bolzano. (PDF) [file pgen.1003534.s011.pdf]

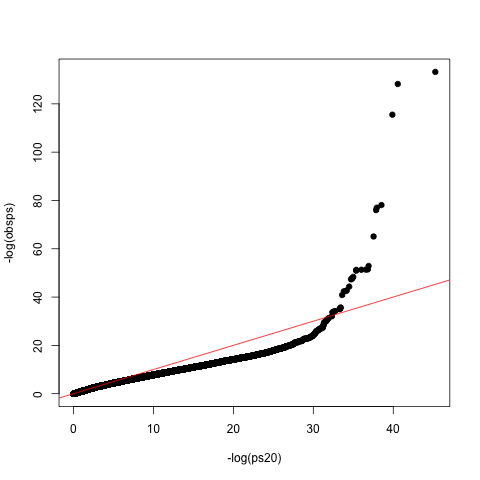

Supplement: Figure S12 — QQ-plot for combined analysis. Quantile-quantile plot for observed p-values from the joint analysis and the simulated distribution obtained under the null (obtained using an alpha value of 20; see text for details). The plot shows the–log10 p-values. (PNG) [file pgen.1003534.s012.png]
