# Supplementary material for: A Genome-Wide, Fine-Scale Map of Natural Pigmentation Variation in Drosophila melanogaster
Source: PLoS Genet. 2013 Jun 6;9(6):e1003534. doi: 10.1371/journal.pgen.1003534 (PMC3674992; doi:10.1371/journal.pgen.1003534)
Supplement: Figure S5 — Receiver operator curves (ROC) for simulations with 10 causal loci. The true and false positive rates are shown on the y- and x- axes, respectively, for the case in which there are 10 causal loci and different genetic penetrance (g), with 25% (top row), 50% (middle row) of 100% of the phenotypic value of an individual due to its genotype. Cases where alleles have uniform effect are denoted with Eff = U, and where alleles have exponentially distributed effects with Eff = E. Causal SNPs were either drawn randomly (mf = 0), or required to have a minor allele frequency of 0.2 (mf = 0.2). The black lines show the cases where extreme phenotypes were selected, and the grey lines show where one extreme phenotype was compared to a random sample (as in a case-control experiment). The solid lines show the same results, except that here regions of zero recombination were excluded from the analysis. Each curve was estimated from 50 simulations. See Text S4 for detailed simulation methods. (PDF) [file pgen.1003534.s005.pdf]

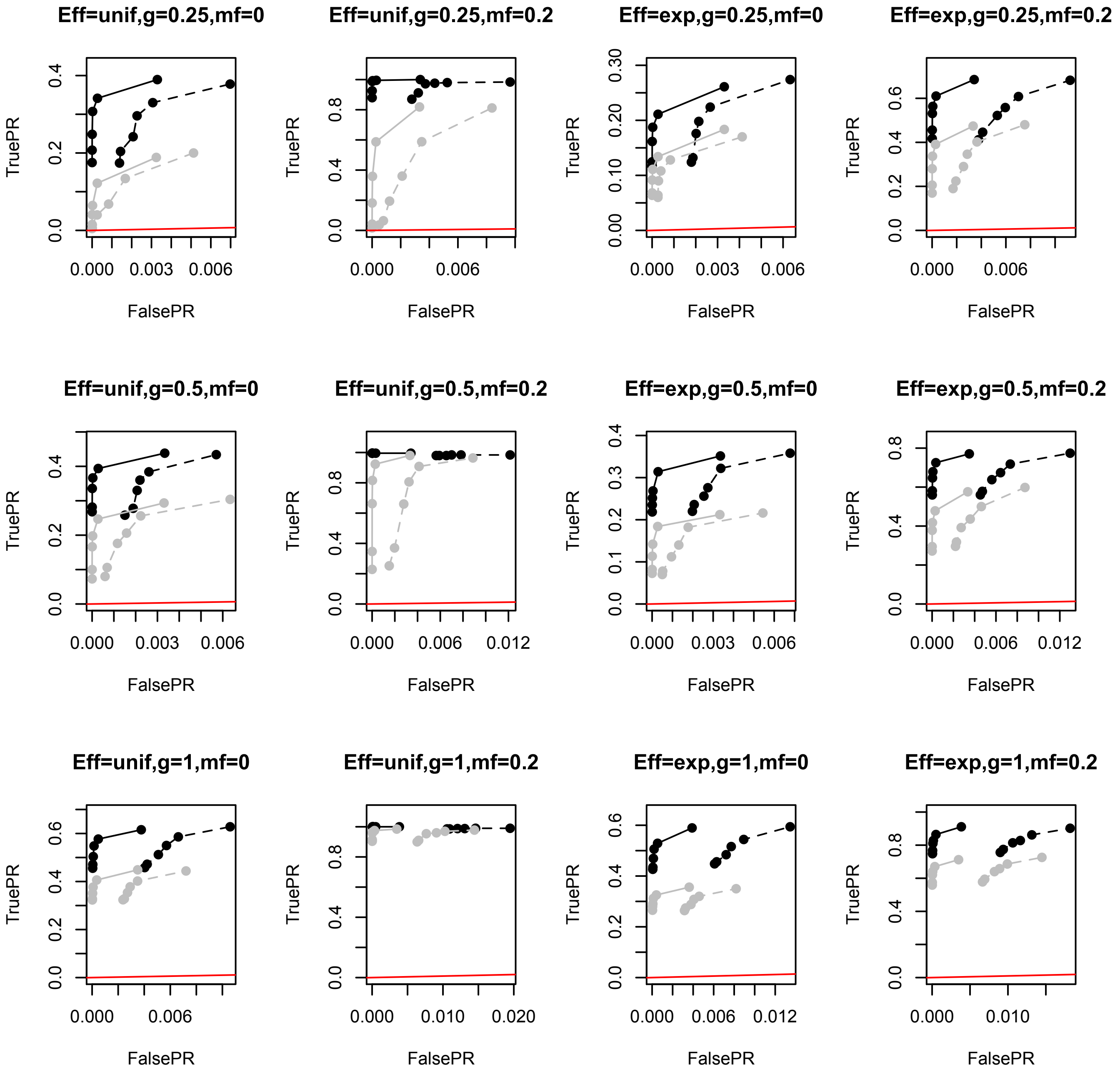
Eff=unif,g=0.25,mf=0
Eff=unif,g=0.25,mf=0.2
Eff=exp,g=0.25,mf=0
Eff=exp,g=0.25,mf=0.2
Eff=unif,g=0.5,mf=0
Eff=unif,g=0.5,mf=0.2
Eff=exp,g=0.5,mf=0
Eff=exp,g=0.5,mf=0.2
Eff=unif,g=1,mf=0
Eff=unif,g=1,mf=0.2
Eff=exp,g=1,mf=0
Eff=exp,g=1,mf=0.2
TruePR
FalsePR
0.0
0.2
0.4
0.6
0.8
0.00
0.10
0.20
0.30
0.1
0.3
0.000
0.003
0.006
0.010
0.012
0.020
