# Supplementary material for: A Genome-Wide, Fine-Scale Map of Natural Pigmentation Variation in Drosophila melanogaster
Source: PLoS Genet. 2013 Jun 6;9(6):e1003534. doi: 10.1371/journal.pgen.1003534 (PMC3674992; doi:10.1371/journal.pgen.1003534)
Supplement: Figure S6 — Receiver operator curves (ROC) for simulations with 20 causal loci. Plots are as before, but summarize simulations in which there were 20 causal loci. (PDF) [file pgen.1003534.s006.pdf]

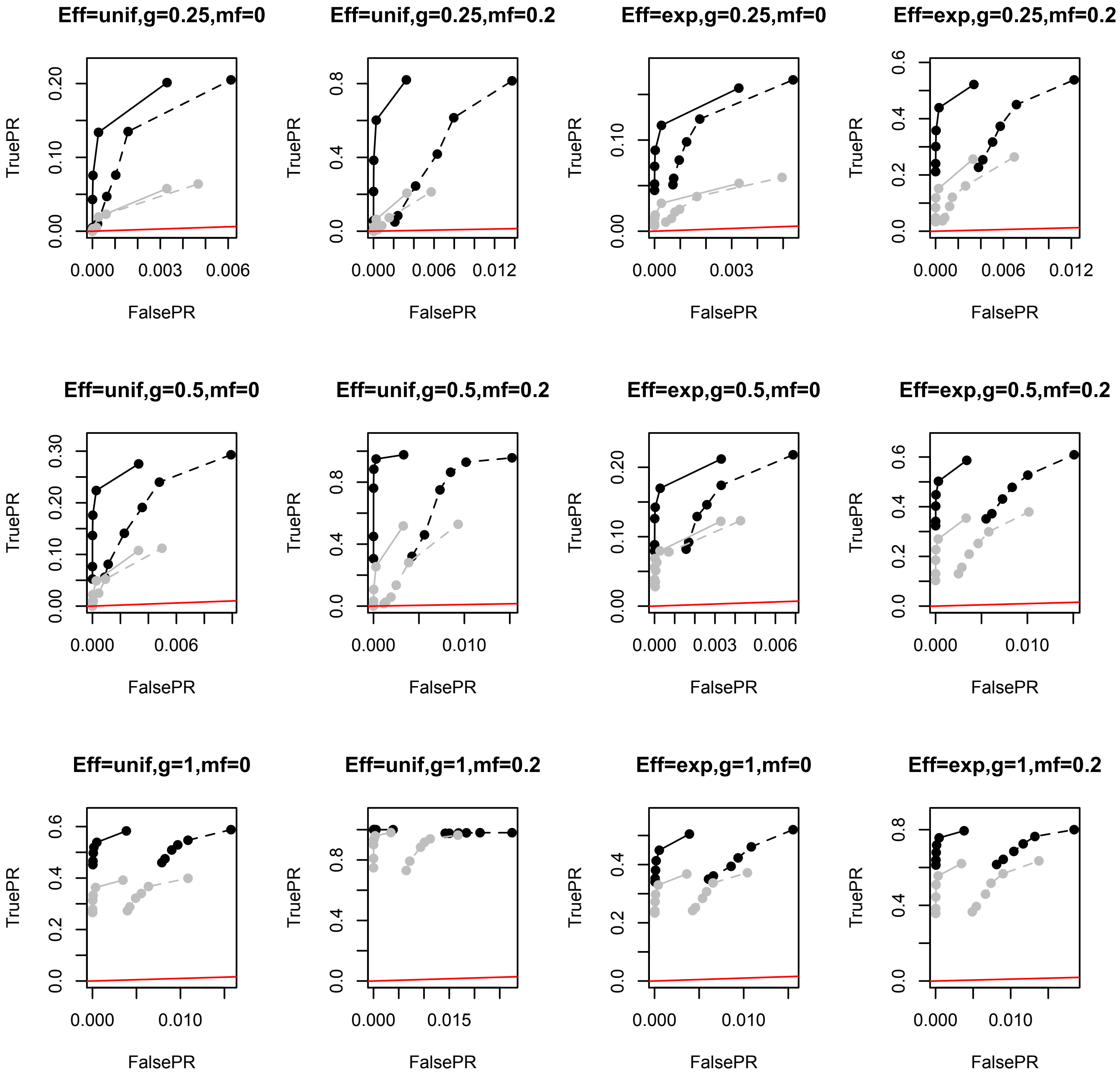

Eff=unif,g=0.25,mf=0
Eff=unif,g=0.25,mf=0.2
Eff=exp,g=0.25,mf=0
Eff=exp,g=0.25,mf=0.2
Eff=unif,g=0.5,mf=0
Eff=unif,g=0.5,mf=0.2
Eff=exp,g=0.5,mf=0
Eff=exp,g=0.5,mf=0.2
Eff=unif,g=1,mf=0
Eff=unif,g=1,mf=0.2
Eff=exp,g=1,mf=0
Eff=exp,g=1,mf=0.2
TruePR
FalsePR
0.00
0.10
0.20
0.000
0.003
0.006
0.0
0.4
0.8
0.012
0.2
0.6
0.30
0.010
0.015
