# Supplementary material for: A Genome-Wide, Fine-Scale Map of Natural Pigmentation Variation in Drosophila melanogaster
Source: PLoS Genet. 2013 Jun 6;9(6):e1003534. doi: 10.1371/journal.pgen.1003534 (PMC3674992; doi:10.1371/journal.pgen.1003534)
Supplement: Figure S7 — Receiver operator curves (ROC) for simulations with 100 causal loci. Plots are as before, but summarize simulations in which there were 100 causal loci. (PDF) [file pgen.1003534.s007.pdf]

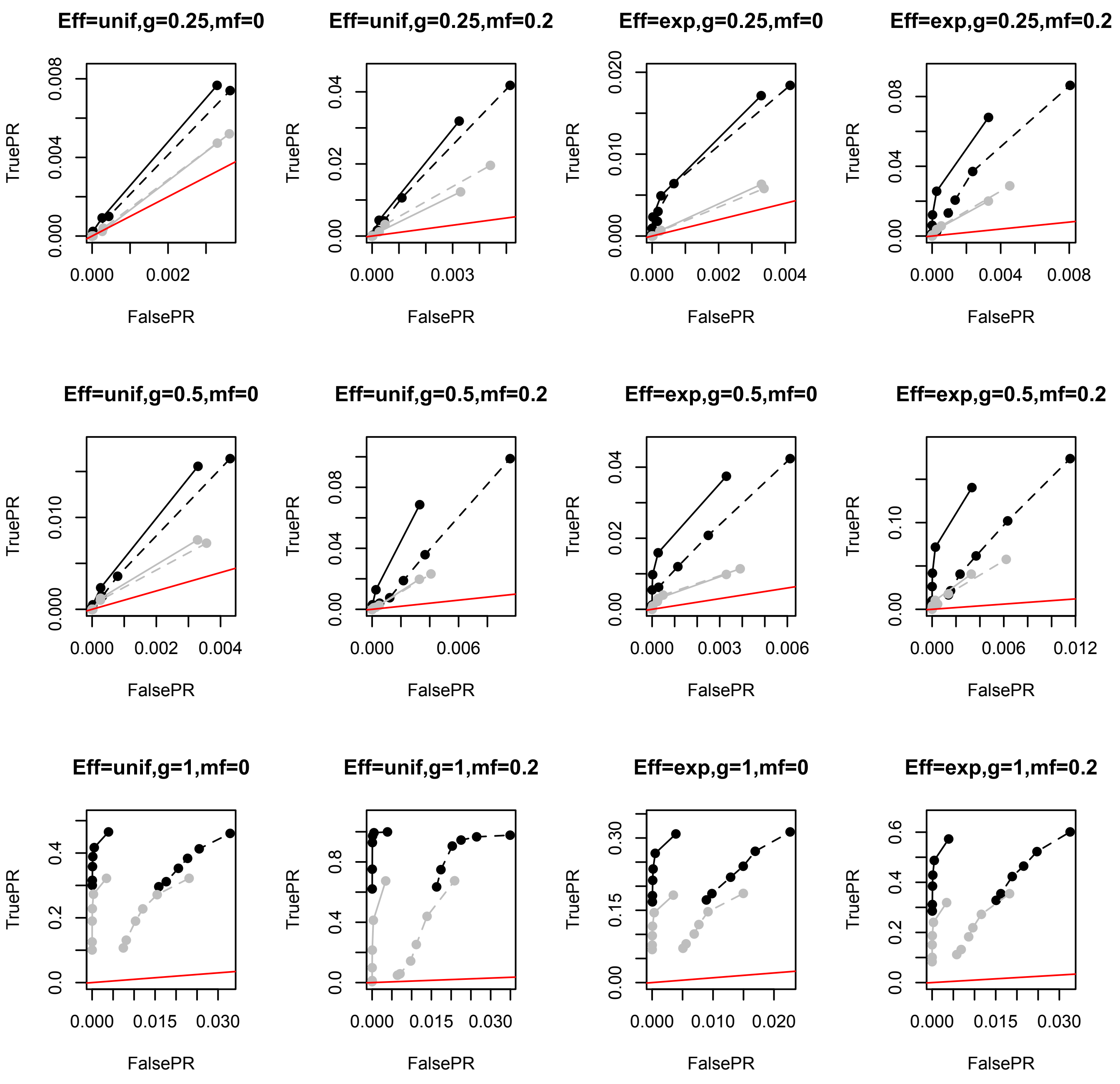
Eff=unif,g=0.25,mf=0
Eff=unif,g=0.25,mf=0.2
Eff=exp,g=0.25,mf=0
Eff=exp,g=0.25,mf=0.2
Eff=unif,g=0.5,mf=0
Eff=unif,g=0.5,mf=0.2
Eff=exp,g=0.5,mf=0
Eff=exp,g=0.5,mf=0.2
Eff=unif,g=1,mf=0
Eff=unif,g=1,mf=0.2
Eff=exp,g=1,mf=0
Eff=exp,g=1,mf=0.2
TruePR
FalsePR
