# Supplementary material for: A Genome-Wide, Fine-Scale Map of Natural Pigmentation Variation in Drosophila melanogaster
Source: PLoS Genet. 2013 Jun 6;9(6):e1003534. doi: 10.1371/journal.pgen.1003534 (PMC3674992; doi:10.1371/journal.pgen.1003534)
Supplement: Table S1 — Characteristics of highly ranked SNPs in the joint analysis of the Viennese and Bolzano samples. For the top 100 ranked SNPs, the table shows the rank, the location (chromosome and position), the reference and alternative nucleotide, the gene(s) in which they lie (gene ID and gene name), the effect on the gene (synonymous coding, intron, etc.), the change in amino acid and codon (if in coding sequence) and the p-value from our analysis. The colors indicate which, if any, pigmentation gene the SNP is near (blue: tan; red: bab1; yellow: ebony; no color: none). (PDF) [file pgen.1003534.s013.pdf]

Supplementary Table 1. SNPs ranked by their p-values in combined analysis of the Viennese and Bolzano populations.

Rank	Chromosome	Position	Reference	Change	Gene ID	Gene name	Effect		Old AA/new AA	Old codon/new codon	*P*-value
1	X	9121129	C	T	FBgn0030108	*Gr8a*	UPSTREAM	41 bases	na	na	1.38804E-58
2	X	9121094	T	C	FBgn0030108	*Gr8a*	UPSTREAM	76 bases	na	na	2.06137E-56
3	X	9120922	G	A	na	*na*	INTERGENIC	na	na	na	6.83878E-51
4	X	9121177	C	T	FBgn0030108	*Gr8a*	UTR 5 PRIME	na	na	na	1.20335E-34
5	X	9121338	A	G	FBgn0030108	*Gr8a*	NON SYNONYMOUS CODING	na	H/R	cAt/cGt	3.63258E-34
6	X	9121191	C	T	FBgn0030108	*Gr8a*	UTR 5 PRIME	na	na	na	9.81618E-34
7	X	9119071	T	G	na	*na*	INTERGENIC	na	na	na	5.41522E-29
8	X	9120204	A	C	FBgn0030107	*CG15370*	NON SYNONYMOUS CODING	na	H/P	cAc/cCc	1.09994E-23
9	X	9119160	T	A	na	*na*	INTERGENIC	na	na	na	4.45855E-23
10	X	9119116	G	A	na	*na*	INTERGENIC	na	na	na	4.9969E-23
11	X	9121584	G	C	FBgn0030108	*Gr8a*	NON SYNONYMOUS CODING	na	G/A	gGc/gCc	5.18733E-23
12	X	9119157	T	C	na	*na*	INTERGENIC	na	na	na	5.55757E-23
13	3L	1085454	T	C	FBgn0004870	*bab1*	INTRON	na	na	na	7.62941E-23
14	X	9118408	T	C	na	*na*	INTERGENIC	na	na	na	9.93985E-22
15	X	9120730	G	C	FBgn0030107	*CG15370*	DOWNSTREAM	129 bases	na	na	1.96712E-21
16	X	9117751	C	T	na	*na*	INTERGENIC	na	na	na	2.1077E-21
17	X	9120683	A	C	FBgn0030107	*CG15370*	DOWNSTREAM	82 bases	na	na	2.78451E-21
18	3L	1074985	G	C	FBgn0004870	*bab1*	INTRON	na	na	na	5.52768E-20
19	X	9123903	A	T	FBgn0030109	*CG12121*	INTRON	na	na	na	2.80031E-19
20	X	9117728	A	T	na	*na*	INTERGENIC	na	na	na	3.38262E-19
21	X	9116599	C	T	FBgn0086367	*t*	INTRON	na	na	na	3.95465E-19
22	3L	1074986	T	G	FBgn0004870	*bab1*	INTRON	na	na	na	1.79784E-18
23	X	9123893	G	A	FBgn0030109	*CG12121*	INTRON	na	na	na	3.09999E-16
24	X	9120247	G	C	FBgn0030107	*CG15370*	SYNONYMOUS CODING	na	R/R	cgG/cgC	4.26437E-16
25	X	9123892	A	C	FBgn0030109	*CG12121*	INTRON	na	na	na	4.77851E-16
26	3L	1102745	G	T	na	*na*	INTERGENIC	na	na	na	6.33641E-16
27	3L	1102996	C	T	na	*na*	INTERGENIC	na	na	na	1.41572E-15
28	3R	17064232	C	A	na	*na*	INTERGENIC	na	na	na	1.53166E-15
29	X	9127299	G	A	FBgn0052704	*Ir8a*	SYNONYMOUS CODING	na	G/G	ggC/ggT	2.1325E-15
30	X	9127794	C	G	FBgn0052704	*Ir8a*	SYNONYMOUS CODING	na	V/V	gtG/gtC	2.33085E-15
31	X	9125001	G	T	FBgn0030109	*CG12121*	NON SYNONYMOUS CODING	na	A/D	gCc/gAc	1.20985E-14
32	X	9117680	G	A	na	*na*	INTERGENIC	na	na	na	2.50336E-14
33	3L	1102726	A	G	na	*na*	INTERGENIC	na	na	na	3.72131E-14
34	X	9121477	G	A	FBgn0030108	*Gr8a*	SYNONYMOUS CODING	na	L/L	ctG/ctA	5.39319E-14
35	X	9113143	A	G	FBgn0086367	*t*	SYNONYMOUS CODING	na	S/S	agT/agC	5.4196E-14
36	X	9122048	A	C	FBgn0030108	*Gr8a*	NON SYNONYMOUS CODING	na	K/Q	Aaa/Caa	8.96889E-14
37	3L	1103020	G	T	na	*na*	INTERGENIC	na	na	na	9.25067E-14
38	3L	1102833	T	C	na	*na*	INTERGENIC	na	na	na	1.37517E-13
39	3L	1103414	T	C	na	*na*	INTERGENIC	na	na	na	1.94274E-13
40	X	9117334	A	C	FBgn0086367	*t*	UPSTREAM	44 bases	na	na	2.97377E-13
41	3L	1074701	G	A	FBgn0004870	*bab1*	INTRON	na	na	na	5.8389E-13
42	X	9119334	T	A	FBgn0030107	*CG15370*	UPSTREAM	155 bases	na	na	6.5942E-13
43	X	9127962	G	A	FBgn0052704	*Ir8a*	SYNONYMOUS CODING	na	I/I	atC/atT	1.23671E-12
44	X	9119872	G	T	FBgn0030107	*CG15370*	SYNONYMOUS CODING	na	P/P	ccG/ccT	1.27094E-12
45	3L	1260112	G	A	FBgn0035199	*CG9134*	INTRON	na	na	na	1.2789E-12
46	3L	1090584	C	A	FBgn0004870	*bab1*	INTRON	na	na	na	1.4283E-12
47	X	9127383	C	T	FBgn0052704	*Ir8a*	SYNONYMOUS CODING	na	K/K	aaG/aaA	1.79946E-12
48	3L	21912511	A	C	FBgn0262737	*mub*	INTRON	na	na	na	1.80077E-12
49	X	9167319	T	C	FBgn0030122	*CG16892*	UPSTREAM	131 bases	na	na	1.83919E-12
50	X	9117516	T	G	na	*na*	INTERGENIC	na	na	na	1.98843E-12
51	3L	1085687	G	A	FBgn0004870	*bab1*	INTRON	na	na	na	2.46326E-12
52	3L	1102064	A	G	na	*na*	INTERGENIC	na	na	na	2.94692E-12
53	3L	1103402	A	T	na	*na*	INTERGENIC	na	na	na	3.18829E-12
54	3L	1102065	T	G	na	*na*	INTERGENIC	na	na	na	3.37687E-12
55	X	9242507	G	T	FBgn0085478	*CG34449*	INTRON	na	na	na	3.58302E-12
56	3R	17064002	A	G	na	*na*	INTERGENIC	na	na	na	3.67442E-12
57	X	9127926	C	T	FBgn0052704	*Ir8a*	SYNONYMOUS CODING	na	R/R	cgG/cgA	4.01758E-12
58	X	9113092	G	A	FBgn0086367	*t*	SYNONYMOUS CODING	na	L/L	ctC/ctT	5.29601E-12
59	X	9113122	G	A	FBgn0086367	*t*	SYNONYMOUS CODING	na	Y/Y	taC/taT	5.3943E-12
60	X	9119866	T	A	FBgn0030107	*CG15370*	SYNONYMOUS CODING	na	A/A	gcT/gcA	5.65005E-12
61	X	9118951	C	G	na	*na*	INTERGENIC	na	na	na	8.85986E-12
62	X	9225280	C	T	FBgn0085478	*CG34449*	DOWNSTREAM / INTRON / UTR 3 PRIME	92 bases / na / na	na	na	9.71941E-12
63	3L	1086181	G	A	FBgn0004870	*bab1*	INTRON	na	na	na	1.11435E-11
64	X	9166681	T	A	FBgn0030121	*CG17446*	INTRON	na	na	na	1.12959E-11
65	X	9119265	G	A	na	*na*	INTERGENIC	na	na	na	1.34807E-11
66	X	9118969	A	G	na	*na*	INTERGENIC	na	na	na	1.62516E-11
67	3L	1074818	C	T	FBgn0004870	*bab1*	INTRON	na	na	na	1.87887E-11
68	X	9125894	T	C	FBgn0030109	*CG12121*	SYNONYMOUS CODING	na	K/K	aaA/aaG	2.08285E-11
69	X	9099953	A	G	FBgn0030102	*CG12119*	DOWNSTREAM	200 bases	na	na	2.20228E-11
70	X	9119833	C	T	FBgn0030107	*CG15370*	SYNONYMOUS CODING	na	D/D	gaC/gaT	2.78556E-11
71	3L	1084059	T	C	FBgn0004870	*bab1*	INTRON	na	na	na	3.12982E-11
72	3L	672619	T	C	na	*na*	INTERGENIC	na	na	na	3.52791E-11
73	X	9113083	A	G	FBgn0086367	*t*	SYNONYMOUS CODING	na	A/A	gcT/gcC	3.95643E-11
74	3L	1102200	T	A	na	*na*	INTERGENIC	na	na	na	4.15024E-11
75	3L	993824	G	T	FBgn0035179	CG12038	UPSTREAM	164 bases	na	na	4.15224E-11
76	X	8832027	A	G	FBgn0261549	*rdgA*	INTRON	na	na	na	4.35254E-11
77	X	9121801	T	C	FBgn0030108	*Gr8a*	SYNONYMOUS CODING	na	H/H	caT/caC	5.61235E-11
78	X	9132456	C	T	FBgn0260789	mxc	SYNONYMOUS CODING	na	A/A	gcG/gcA	5.63008E-11
79	X	9125150	T	C	FBgn0030109	*CG12121*	SYNONYMOUS CODING	na	E/E	gaA/gaG	6.25503E-11
80	X	9113622	A	G	FBgn0086367	*t*	INTRON	na	na	na	6.41577E-11
81	3R	12742092	G	A	FBgn0262906	*msa*	INTRON	na	na	na	7.41026E-11
82	X	9168413	A	C	FBgn0030122	*CG16892*	SYNONYMOUS CODING	na	S/S	tcA/tcC	7.66433E-11
83	X	8882814	T	G	FBgn0030073	*CG10962 / rdgA*	INTRON	na	na	na	8.05017E-11
84	3L	1103291	T	A	na	*na*	INTERGENIC	na	na	na	8.66115E-11
85	X	9116537	A	G	FBgn0086367	*t*	INTRON	na	na	na	9.59672E-11
86	X	9167423	A	T	FBgn0030122	*CG16892*	UPSTREAM	27 bases	na	na	1.02723E-10
87	X	9113116	A	C	FBgn0086367	*t*	SYNONYMOUS CODING	na	V/V	gtT/gtG	1.03401E-10
88	3L	7183480	C	T	na	*na*	INTERGENIC	na	na	na	1.03464E-10
89	3L	1103300	T	G	na	*na*	INTERGENIC	na	na	na	1.14197E-10
90	X	9686968	T	A	FBgn0052698	*CG32698*	INTRON	na	na	na	1.25269E-10
91	X	9200486	C	T	na	*na*	INTERGENIC	na	na	na	1.25917E-10
92	2R	4383278	C	T	FBgn0050361	*mtt*	INTRON	na	na	na	1.3051E-10
93	3L	1972518	C	A	FBgn0035300	*CG1139*	SYNONYMOUS CODING	na	P/P	ccC/ccA	1.33466E-10
94	3L	4916850	A	T	FBgn0028699	*Rh50*	INTRON	na	na	na	1.34815E-10
95	3L	1104190	C	T	na	*na*	INTERGENIC	na	na	na	1.37099E-10
96	X	9125615	G	A	FBgn0030109	*CG12121*	INTRON	na	na	na	1.38642E-10
97	X	9965042	C	T	FBgn0030174	*CG15312*	UPSTREAM	143 bases	na	na	1.50068E-10
98	X	9125023	C	G	FBgn0030109	*CG12121*	NON SYNONYMOUS CODING	na	E/Q	Gaa/Caa	1.53996E-10
99	X	9200484	A	C	na	*na*	INTERGENIC	na	na	na	1.63982E-10
100	3L	1102221	T	G	na	*na*	INTERGENIC	na	na	na	1.66088E-10
