# Supplementary material for: A Genome-Wide, Fine-Scale Map of Natural Pigmentation Variation in Drosophila melanogaster
Source: PLoS Genet. 2013 Jun 6;9(6):e1003534. doi: 10.1371/journal.pgen.1003534 (PMC3674992; doi:10.1371/journal.pgen.1003534)
Supplement: Table S2 — Characteristics of highly ranked SNPs in the analysis of the Viennese sample analyzed alone. Values given are as in Table S1, except that the ranking corresponds to the ranking in the Viennese sample. (PDF) [file pgen.1003534.s014.pdf]

Supplementary Table 2. SNPs ranked by the p-values in the analysis of the Viennese sample.

Rank	Chromosome	Position	Reference	Change	Gene ID	Gene name	Effect		Old AA/new AA	Old codon/new codon	*P*-value
1	X	9121094	T	C	FBgn0030108	*Gr8a*	UPSTREAM	76 bases	na	na	2.10998E-18
2	X	9120922	G	A	na	*na*	INTERGENIC	na	na	na	2.31946E-17
3	X	9121129	C	T	FBgn0030108	*Gr8a*	UPSTREAM	41 bases	na	na	4.19516E-15
4	X	9121177	C	T	FBgn0030108	*Gr8a*	UTR 5 PRIME	na	na	na	1.66829E-13
5	X	9121191	C	T	FBgn0030108	*Gr8a*	UTR 5 PRIME	na	na	na	4.38058E-13
6	3L	21912511	A	C	FBgn0262737	*mub*	INTRON	na	na	na	1.80077E-12
7	X	9119071	T	G	na	*na*	INTERGENIC	na	na	na	7.77332E-12
8	X	9119573	T	C	FBgn0030107	*CG15370*	SYNONYMOUS CODING	na	L/L	Ttg/Ctg	2.44355E-11
9	X	9126564	A	C	FBgn0030109 / FBgn0052704	*CG12121 / Ir8a*	UPSTREAM / DOWNSTREAM	158 bases / 195 bases	na	na	6.06574E-11
10	3L	1074985	G	C	FBgn0004870	*bab1*	INTRON	na	na	na	7.00211E-11
11	X	9120683	A	C	FBgn0030107	*CG15370*	DOWNSTREAM	82 bases	na	na	5.50451E-10
12	X	9119566	A	G	FBgn0030107	*CG15370*	SYNONYMOUS CODING	na	L/L	ttA/ttG	7.31331E-10
13	3R	9719690	A	T	na	*na*	INTERGENIC	na	na	na	9.38569E-10
14	3L	1074986	T	G	FBgn0004870	*bab1*	INTRON	na	na	na	9.6799E-10
15	3R	17064232	C	A	na	*na*	INTERGENIC	na	na	na	1.14696E-09
16	X	9119525	G	T	FBgn0030107	*CG15370*	NON SYNONYMOUS CODING	na	G/C	Ggc/Tgc	1.17153E-09
17	3L	993824	G	T	FBgn0035179	*CG12038*	UPSTREAM	164 bases	na	na	7.1538E-09
18	3L	16459331	A	C	FBgn0053158	*CG33158*	INTRON	na	na	na	7.16885E-09
19	3R	20246946	A	G	FBgn0000036	*nAcRalpha-96Aa*	INTRON	na	na	na	8.08072E-09
20	X	8093740	C	A	FBgn0261873	*sdt*	INTRON	na	na	na	8.65304E-09
21	3R	19775580	C	T	na	*na*	INTERGENIC	na	na	na	1.20787E-08
22	X	9119116	G	A	na	*na*	INTERGENIC	na	na	na	1.28688E-08
23	3L	3997530	A	T	na	*na*	INTERGENIC	na	na	na	1.53473E-08
24	3L	16183821	A	C	na	*na*	INTERGENIC	na	na	na	1.84928E-08
25	2R	12736666	A	G	na	*na*	INTERGENIC	na	na	na	1.87135E-08
26	3R	15674057	T	G	FBgn0038735	*CG4662*	INTRON	na	na	na	2.46079E-08
27	3L	1102064	A	G	na	*na*	INTERGENIC	na	na	na	2.68421E-08
28	3R	21502989	T	A	FBgn0039354 / FBgn0039355	*Lgr3 / CG4730*	DOWNSTREAM / UTR 3 PRIME	51 bases / na	na	na	2.85977E-08
29	3R	18389434	T	C	na	*na*	INTERGENIC	na	na	na	3.48683E-08
30	3L	11195204	G	A	FBgn0036146 / FBgn0036147	*CG14141 / Plod*	UTR 3 PRIME / INTRON	na	na	na	3.9388E-08
31	3L	12374579	G	A	FBgn0036279	*Ncc69*	INTRON	na	na	na	4.31349E-08
32	X	3156275	A	G	FBgn0000479	*dnc*	INTRON	na	na	na	5.23554E-08
33	2R	17536263	G	T	FBgn0034644	*CG10082*	INTRON	na	na	na	5.62448E-08
34	2R	6447557	G	A	FBgn0263102	*psq*	INTRON	na	na	na	5.68251E-08
35	3L	7775673	C	T	FBgn0052369	*CG32369*	INTRON	na	na	na	6.267E-08
36	2L	13148294	A	T	na	*na*	INTERGENIC	na	na	na	6.84069E-08
37	2L	11201295	G	T	na	*na*	INTERGENIC	na	na	na	6.90847E-08
38	3L	1882504	A	G	FBgn0035287	*CG13937*	INTRON / UTR 5 PRIME	na	na	na	7.25606E-08
39	2L	16720014	G	T	FBgn0000409 / FBgn0051782	*Cyt-c-p / CG31782*	INTRON	na	na	na	7.56056E-08
40	3L	1102726	A	G	na	*na*	INTERGENIC	na	na	na	7.65391E-08
41	3L	20697525	A	T	na	*na*	INTERGENIC	na	na	na	8.63638E-08
42	2R	12176768	C	T	FBgn0028424	*JhI-26*	UPSTREAM	62 bases	na	na	8.71092E-08
43	3L	12374818	C	T	FBgn0036279	*Ncc69*	INTRON	na	na	na	9.22663E-08
44	2R	4271816	A	G	FBgn0261588	*pdm3*	INTRON	na	na	na	1.00241E-07
45	2L	5096101	A	T	FBgn0031683	*CG4230*	UPSTREAM	126 bases	na	na	1.04088E-07
46	2L	5928790	T	G	FBgn0015381	*dsf*	INTRON	na	na	na	1.11565E-07
47	2L	16692566	A	T	FBgn0261278	*grp*	INTRON	na	na	na	1.26709E-07
48	3L	1102065	T	G	na	*na*	INTERGENIC	na	na	na	1.30614E-07
49	X	9686968	T	A	FBgn0052698	*CG32698*	INTRON	na	na	na	1.32812E-07
50	2R	3024590	G	C	na	*na*	INTERGENIC	na	na	na	1.33271E-07
51	3R	20422142	C	T	FBgn0083077	*mld*	INTRON	na	na	na	1.33443E-07
52	3R	14016344	T	C	FBgn0038578	*MED17*	SYNONYMOUS CODING	na	H/H	caT/caC	1.37713E-07
53	X	9103364	G	A	FBgn0030102	*CG12119*	INTRON	na	na	na	1.40708E-07
54	3R	18389442	A	G	na	*na*	INTERGENIC	na	na	na	1.43678E-07
55	X	9202554	T	A	na	*na*	INTERGENIC	na	na	na	1.4954E-07
56	3L	12374566	G	T	FBgn0036279	*Ncc69*	INTRON	na	na	na	1.58216E-07
57	X	9116089	G	T	FBgn0086367	*t*	INTRON	na	na	na	1.63587E-07
58	3L	1151546	A	G	FBgn0025525	*bab2*	INTRON	na	na	na	1.6651E-07
59	3R	18389441	T	A	na	*na*	INTERGENIC	na	na	na	1.8344E-07
60	3R	16414118	G	A	FBgn0014029	*39692*	UPSTREAM	33 bases	na	na	1.83486E-07
61	2R	20200863	T	A	na	*na*	INTERGENIC	na	na	na	2.00722E-07
62	2L	16622940	C	A	FBgn0259735	*CG42389*	INTRON	na	na	na	2.1505E-07
63	3R	9486452	G	A	na	*na*	INTERGENIC	na	na	na	2.2062E-07
64	2L	16828016	T	G	FBgn0051739	*CG31739*	INTRON	na	na	na	2.33503E-07
65	3R	14580485	A	G	FBgn0038651	*CG14299*	SYNONYMOUS CODING	na	F/F	ttT/ttC	2.33762E-07
66	3R	21420180	G	A	FBgn0011666 / FBgn0011666 / FBgn0261287	*msi / msi / ymp*	INTRON / UPSTREAM / INTRON	na / 55 bases / na	na	na	2.34102E-07
67	2L	15758500	G	A	FBgn0001987	*Gli*	SYNONYMOUS CODING	na	I/I	atC/atT	2.35741E-07
68	3L	11086124	T	A	FBgn0020412	*JIL-1*	UTR 5 PRIME	na	na	na	2.40967E-07
69	2R	20171916	T	C	FBgn0005636	*nvy*	INTRON	na	na	na	2.48411E-07
70	2L	6007626	T	A	FBgn0031762	*CG9098*	UTR 3 PRIME	na	na	na	2.59722E-07
71	X	9110738	G	A	FBgn0030105	*CG15369*	UPSTREAM	188 bases	na	na	2.69622E-07
72	2R	14353775	G	A	FBgn0028496	*CG30116*	INTRON	na	na	na	2.82239E-07
73	3L	1102745	G	T	na	*na*	INTERGENIC	na	na	na	2.98018E-07
74	X	6555533	T	C	FBgn0029885 / FBgn0062413 / FBgn0062413	*CG3224 / Ctr1A / Ctr1A*	UPSTREAM	53 bases / 147 bases / 149 bases	na	na	3.05093E-07
75	X	12942344	A	C	FBgn0261388	*CG42629*	INTRON	na	na	na	3.08455E-07
76	3R	5324494	A	T	FBgn0014380	*RhoL*	INTRON	na	na	na	3.08736E-07
77	3R	21388535	T	A	FBgn0011666	*msi*	INTRON	na	na	na	3.10419E-07
78	X	571471	T	C	na	*na*	INTERGENIC	na	na	na	3.10446E-07
79	2L	2689324	C	A	FBgn0051690	*CG31690*	INTRON	na	na	na	3.2341E-07
80	X	9121477	G	A	FBgn0030108	*Gr8a*	SYNONYMOUS CODING	na	L/L	ctG/ctA	3.3765E-07
81	2L	10134230	C	G	FBgn0262032	*CG42843*	INTRON	na	na	na	3.45653E-07
82	2L	7937813	C	A	FBgn0085450	*Snoo*	INTRON	na	na	na	3.72785E-07
83	2R	11671706	C	T	FBgn0083919	*Zasp52*	INTRON	na	na	na	3.81057E-07
84	3R	16176849	C	G	na	*na*	INTERGENIC	na	na	na	3.85121E-07
85	3R	12632306	G	C	na	*na*	INTERGENIC	na	na	na	3.86794E-07
86	3L	487973	G	A	FBgn0001316	*klar*	INTRON	na	na	na	3.8828E-07
87	X	9119334	T	A	FBgn0030107	*CG15370*	UPSTREAM	155 bases	na	na	3.97832E-07
88	3L	4156817	G	C	FBgn0259986	*nab*	INTRON	na	na	na	4.0824E-07
89	3L	16183832	T	C	na	*na*	INTERGENIC	na	na	na	4.13235E-07
90	3R	11699474	T	G	na	*na*	INTERGENIC	na	na	na	4.27119E-07
91	3L	1084059	T	C	FBgn0004870	*bab1*	INTRON	na	na	na	4.36772E-07
92	2L	18336626	C	T	FBgn0000636	*Fas3*	INTRON	na	na	na	4.45686E-07
93	3R	15426383	G	A	FBgn0038715	*CG7333*	UTR 5 PRIME	na	na	na	4.45704E-07
94	2R	16602275	A	C	FBgn0040726	*dpr*	INTRON	na	na	na	4.70335E-07
95	2L	19270799	G	T	na	*na*	INTERGENIC	na	na	na	4.97044E-07
96	3L	14450691	C	A	FBgn0087007	*bbg*	INTRON	na	na	na	5.06816E-07
97	3R	24764585	C	T	FBgn0039617	*CG14521*	INTRON	na	na	na	5.13086E-07
98	3R	9614278	G	A	na	*na*	INTERGENIC	na	na	na	5.16677E-07
99	3R	17205366	A	G	FBgn0004110	*tin*	INTRON	na	na	na	5.18389E-07
100	3R	14704756	A	T	na	*na*	INTERGENIC	na	na	na	5.23691E-07
