# Supplementary material for: A Genome-Wide, Fine-Scale Map of Natural Pigmentation Variation in Drosophila melanogaster
Source: PLoS Genet. 2013 Jun 6;9(6):e1003534. doi: 10.1371/journal.pgen.1003534 (PMC3674992; doi:10.1371/journal.pgen.1003534)
Supplement: Table S3 — Characteristics of highly ranked SNPs in the analysis of the Bolzano sample analyzed alone. Values given are as in Table S1, except that the ranking corresponds to the ranking in the Bolzano sample. (PDF) [file pgen.1003534.s015.pdf]

Supplementary Table 3. SNPs ranked by the p-values in the analysis of the Bolzano sample.

Rank	Chromosome	Position	Reference	Change	Gene ID	Gene name	Effect		Old AA/new AA	Old codon/new codon	*P*-value
1	X	9121129	C	T	FBgn0030108	*Gr8a*	UPSTREAM	41 bases	na	na	3.00786E-45
2	X	9121094	T	C	FBgn0030108	*Gr8a*	UPSTREAM	76 bases	na	na	3.3587E-39
3	X	9120922	G	A	na	*na*	INTERGENIC	na	na	na	8.62346E-35
4	X	9121338	A	G	FBgn0030108	*Gr8a*	NON SYNONYMOUS CODING	na	H/R	cAt/cGt	3.18408E-30
5	X	9117751	C	T	na	*na*	INTERGENIC	na	na	na	7.45975E-24
6	X	9120204	A	C	FBgn0030107	*CG15370*	NON SYNONYMOUS CODING	na	H/P	cAc/cCc	1.38873E-22
7	X	9121177	C	T	FBgn0030108	*Gr8a*	UTR 5 PRIME	na	na	na	1.95327E-22
8	X	9121191	C	T	FBgn0030108	*Gr8a*	UTR 5 PRIME	na	na	na	6.2378E-22
9	X	9117728	A	T	na	*na*	INTERGENIC	na	na	na	9.64134E-21
10	3L	1085454	T	C	FBgn0004870	*bab1*	INTRON	na	na	na	3.6021E-20
11	X	9119160	T	A	na	*na*	INTERGENIC	na	na	na	2.91025E-19
12	X	9121584	G	C	FBgn0030108	*Gr8a*	NON SYNONYMOUS CODING	na	G/A	gGc/gCc	3.62114E-19
13	X	9116599	C	T	FBgn0086367	*t*	INTRON	na	na	na	1.13539E-18
14	X	9119071	T	G	na	*na*	INTERGENIC	na	na	na	2.024E-18
15	X	9118408	T	C	na	*na*	INTERGENIC	na	na	na	5.00367E-18
16	X	9119157	T	C	na	*na*	INTERGENIC	na	na	na	8.92825E-18
17	X	9120247	G	C	FBgn0030107	*CG15370*	SYNONYMOUS CODING	na	R/R	cgG/cgC	3.16493E-17
18	X	9120730	G	C	FBgn0030107	*CG15370*	DOWNSTREAM	129 bases	na	na	7.85617E-17
19	X	9127794	C	G	FBgn0052704	*Ir8a*	SYNONYMOUS CODING	na	V/V	gtG/gtC	8.38995E-17
20	X	9127299	G	A	FBgn0052704	*Ir8a*	SYNONYMOUS CODING	na	G/G	ggC/ggT	7.85402E-16
21	X	9119116	G	A	na	*na*	INTERGENIC	na	na	na	1.18722E-15
22	3L	1102996	C	T	na	*na*	INTERGENIC	na	na	na	5.83356E-15
23	X	9489820	G	A	na	*na*	INTERGENIC	na	na	na	9.01864E-15
24	X	9113143	A	G	FBgn0086367	*t*	SYNONYMOUS CODING	na	S/S	agT/agC	1.16727E-14
25	X	9117334	A	C	FBgn0086367	*t*	UPSTREAM	44 bases	na	na	1.25861E-14
26	X	9123903	A	T	FBgn0030109	*CG12121*	INTRON	na	na	na	3.4725E-14
27	X	9116537	A	G	FBgn0086367	*t*	INTRON	na	na	na	5.02189E-14
28	X	9116526	C	T	FBgn0086367	*t*	INTRON	na	na	na	5.24837E-14
29	X	9113122	G	A	FBgn0086367	*t*	SYNONYMOUS CODING	na	Y/Y	taC/taT	6.73531E-14
30	X	9113092	G	A	FBgn0086367	*t*	SYNONYMOUS CODING	na	L/L	ctC/ctT	7.35973E-14
31	X	9127383	C	T	FBgn0052704	*Ir8a*	SYNONYMOUS CODING	na	K/K	aaG/aaA	8.08461E-14
32	3L	4916850	A	T	FBgn0028699	*Rh50*	INTRON	na	na	na	1.42931E-13
33	X	9127926	C	T	FBgn0052704	*Ir8a*	SYNONYMOUS CODING	na	R/R	cgG/cgA	1.84277E-13
34	3L	1103291	T	A	na	*na*	INTERGENIC	na	na	na	3.36151E-13
35	X	9117680	G	A	na	*na*	INTERGENIC	na	na	na	6.38053E-13
36	3L	1103414	T	C	na	*na*	INTERGENIC	na	na	na	1.04177E-12
37	3L	1260112	G	A	FBgn0035199	*CG9134*	INTRON	na	na	na	1.27879E-12
38	X	9120683	A	C	FBgn0030107	*CG15370*	DOWNSTREAM	82 bases	na	na	1.39765E-12
39	X	9125001	G	T	FBgn0030109	*CG12121*	NON SYNONYMOUS CODING	na	A/D	gCc/gAc	1.5797E-12
40	3L	1103020	G	T	na	*na*	INTERGENIC	na	na	na	1.65217E-12
41	3L	1103831	T	C	na	*na*	INTERGENIC	na	na	na	1.86613E-12
42	X	9122048	A	C	FBgn0030108	*Gr8a*	NON SYNONYMOUS CODING	na	K/Q	Aaa/Caa	3.12073E-12
43	X	9113083	A	G	FBgn0086367	*t*	SYNONYMOUS CODING	na	A/A	gcT/gcC	3.52502E-12
44	X	9242507	G	T	FBgn0085478	*CG34449*	INTRON	na	na	na	3.58302E-12
45	3L	1103834	C	T	na	*na*	INTERGENIC	na	na	na	4.16399E-12
46	3L	1102200	T	A	na	*na*	INTERGENIC	na	na	na	4.24158E-12
47	X	9118951	C	G	na	*na*	INTERGENIC	na	na	na	5.6517E-12
48	X	9168413	A	C	FBgn0030122	*CG16892*	SYNONYMOUS CODING	na	S/S	tcA/tcC	6.71948E-12
49	X	9127962	G	A	FBgn0052704	*Ir8a*	SYNONYMOUS CODING	na	I/I	atC/atT	7.12492E-12
50	X	9166681	T	A	FBgn0030121	*CG17446*	INTRON	na	na	na	8.60701E-12
51	3L	7744827	T	G	FBgn0052373	*CG32373*	INTRON	na	na	na	9.40262E-12
52	X	9333825	A	C	FBgn0261260	*Megalin*	INTRON	na	na	na	9.5644E-12
53	3R	17064002	A	G	na	*na*	INTERGENIC	na	na	na	1.14577E-11
54	X	9236567	C	T	FBgn0085478	*CG34449*	INTRON	na	na	na	1.17087E-11
55	X	9100392	C	T	FBgn0030102	*CG12119*	UTR 3 PRIME	na	na	na	1.27708E-11
56	X	9122107	A	C	FBgn0030108	*Gr8a*	SYNONYMOUS CODING	na	I/I	atA/atC	1.99147E-11
57	3L	1103287	A	G	na	*na*	INTERGENIC	na	na	na	2.10812E-11
58	3L	3175541	G	A	FBgn0035410	*CG14964*	SYNONYMOUS CODING	na	S/S	tcC/tcT	2.12315E-11
59	2R	19639341	A	G	FBgn0034901	*CG11300*	UPSTREAM	97 bases	na	na	2.25801E-11
60	X	9118181	A	G	na	*na*	INTERGENIC	na	na	na	3.07674E-11
61	X	9101066	A	G	FBgn0030102	*CG12119*	SYNONYMOUS CODING	na	D/D	gaT/gaC	3.36463E-11
62	X	9200484	A	C	na	*na*	INTERGENIC	na	na	na	3.48154E-11
63	3L	675752	A	G	na	*na*	INTERGENIC	na	na	na	3.50745E-11
64	3L	1088081	G	A	FBgn0004870	*bab1*	INTRON	na	na	na	3.52129E-11
65	X	9200486	C	T	na	*na*	INTERGENIC	na	na	na	3.55397E-11
66	X	9099953	A	G	FBgn0030102	*CG12119*	DOWNSTREAM	200 bases	na	na	3.75606E-11
67	3L	12900701	T	C	na	*na*	INTERGENIC	na	na	na	4.06038E-11
68	3L	1118223	C	T	na	*na*	INTERGENIC	na	na	na	4.3162E-11
69	X	7999141	G	A	FBgn0040319	*Gclc*	SYNONYMOUS CODING	na	F/F	ttC/ttT	4.77102E-11
70	X	9118164	G	T	na	*na*	INTERGENIC	na	na	na	5.71945E-11
71	3L	1103288	A	G	na	*na*	INTERGENIC	na	na	na	5.9726E-11
72	X	9123892	A	C	FBgn0030109	*CG12121*	INTRON	na	na	na	6.42379E-11
73	X	9125150	T	C	FBgn0030109	*CG12121*	SYNONYMOUS CODING	na	E/E	gaA/gaG	6.45131E-11
74	X	9118969	A	G	na	*na*	INTERGENIC	na	na	na	6.65578E-11
75	3L	1103402	A	T	na	*na*	INTERGENIC	na	na	na	7.57954E-11
76	3L	4308517	T	C	na	*na*	INTERGENIC	na	na	na	7.71086E-11
77	X	9205897	G	C	na	*na*	INTERGENIC	na	na	na	7.897E-11
78	3L	1103289	T	A	na	*na*	INTERGENIC	na	na	na	8.08129E-11
79	X	917349	G	A	FBgn0003638 / FBgn0040351 / FBgn0052814	*su(w[a]) / CG11638 / CG32814*	INTRON	na	na	na	8.81683E-11
80	2R	3301871	A	G	na	*na*	INTERGENIC	na	na	na	9.24607E-11
81	3L	1103325	C	T	na	*na*	INTERGENIC	na	na	na	1.0191E-10
82	3L	1074985	G	C	FBgn0004870	*bab1*	INTRON	na	na	na	1.02191E-10
83	X	9298341	T	G	FBgn0261260	*Megalin*	INTRON	na	na	na	1.04227E-10
84	3L	18750653	A	G	FBgn0001078	*ftz-f1*	INTRON	na	na	na	1.08365E-10
85	X	9123893	G	A	FBgn0030109	*CG12121*	INTRON	na	na	na	1.13901E-10
86	3L	18180742	T	G	na	*na*	INTERGENIC	na	na	na	1.22435E-10
87	3L	1118224	G	T	na	*na*	INTERGENIC	na	na	na	1.25115E-10
88	X	8832027	A	G	FBgn0261549	*rdgA*	INTRON	na	na	na	1.28129E-10
89	3L	1090584	C	A	FBgn0004870	*bab1*	INTRON	na	na	na	1.48789E-10
90	3R	13717784	A	T	FBgn0053547	*Rim*	INTRON	na	na	na	1.5934E-10
91	3L	1104181	T	G	na	*na*	INTERGENIC	na	na	na	1.61918E-10
92	3L	12923061	G	A	na	*na*	INTERGENIC	na	na	na	1.62294E-10
93	X	9225280	C	T	FBgn0085478	*CG34449*	DOWNSTREAM / INTRON / UTR 3 PRIME	92 bases / na / na	na	na	1.62824E-10
94	3L	1243534	T	C	FBgn0004378	*Klp61F*	NON SYNONYMOUS CODING	na	M/V	Atg/Gtg	1.79286E-10
95	3L	1103327	G	T	na	*na*	INTERGENIC	na	na	na	1.79975E-10
96	2L	20842105	G	C	FBgn0051676	*CG31676*	INTRON	na	na	na	1.9135E-10
97	3L	4990676	G	A	na	*na*	INTERGENIC	na	na	na	2.02837E-10
98	3L	795644	G	C	na	*na*	INTERGENIC	na	na	na	2.11211E-10
99	3L	15983801	C	T	FBgn0259824	*Hip14*	SYNONYMOUS CODING	na	F/F	ttC/ttT	2.2827E-10
100	3L	4990730	A	G	na	*na*	INTERGENIC	na	na	na	2.587E-10
