# Supplementary material for: A Genome-Wide, Fine-Scale Map of Natural Pigmentation Variation in Drosophila melanogaster
Source: PLoS Genet. 2013 Jun 6;9(6):e1003534. doi: 10.1371/journal.pgen.1003534 (PMC3674992; doi:10.1371/journal.pgen.1003534)
Supplement: Table S4 — Differences in indel frequencies in the regions near the tan, bab1 and ebony loci. These regions are defined, as before, as 20 kb up- and down- stream of the center of the coding sequence of each gene (within 20 kb of the middle of the coding sequence of these genes); indels are found as described in Text S1 with Dindel. As Dindel divides the reads in these regions into windows, some indels occur in more than one window. In these cases, the presence and absence counts of these indels were usually quite similar, and so we give the average coverage and frequency and the minimum and maximum p-values. Similarly, indels with adjacent positions are also merged into a single entry, and the same information is reported for these. (PDF) [file pgen.1003534.s016.pdf]

gene	chrom	pos	ref	total coverage light	total coverage dark	overall frequency light	overall frequency dark	frequency difference	min CMH p-value	max CMH p-value
bab	3L	1071553	ATGT	145.6	138.8	0.93	0.69	0.25	1.11E-07	3.10E-05
bab	3L	1073758	C	156.0	144.0	0.67	0.88	0.22	4.33E-05	
bab	3L	1103421	C	301.5	203.0	0.15	0.35	0.20	5.22E-09	7.37E-05
tan	X	9117778	A	468.5	514.5	0.27	0.07	0.20	3.80E-17	3.29E-15
tan	X	9119088	T	423.0	479.0	0.38	0.19	0.19	3.48E-10	
tan	X	9130925	A	252.0	252.0	0.32	0.13	0.19	3.61E-07	
tan	X	9130932	A	234.0	243.5	0.30	0.11	0.19	3.43E-07	1.66E-06
tan	X	9119093	TT	373.5	429.0	0.26	0.09	0.18	5.62E-11	8.08E-11
tan	X	9119029	G	390.0	483.0	0.29	0.12	0.17	7.41E-10	
bab	3L	1103383	A	268.0	168.0	0.11	0.26	0.15	6.09E-05	
bab	3L	1103380	TG	269.0	166.0	0.11	0.27	0.15	7.32E-05	8.98E-05
tan	X	9116769	TG	385.8	449.8	0.19	0.34	0.15	8.44E-07	3.34E-05
bab	3L	1103251	AG	209.5	147.5	0.11	0.25	0.15	6.32E-05	8.36E-05
bab	3L	1088081	G	414.0	433.0	0.39	0.25	0.14	3.68E-05	
bab	3L	1074810	A	512.0	471.0	0.27	0.13	0.14	7.43E-09	
bab	3L	1072093	A	377.0	412.5	0.70	0.56	0.14	7.29E-05	8.80E-05
bab	3L	1086196	T	399.0	428.0	0.18	0.31	0.13	1.78E-05	
bab	3L	1086189	TT	378.2	426.4	0.63	0.52	0.11	2.83E-08	6.88E-05
tan	X	9106644	TACA	437.8	474.3	0.10	0.01	0.09	3.14E-08	7.06E-08
bab	3L	1085457	C	454.0	457.0	0.09	0.02	0.08	1.18E-06	
bab	3L	1094474	TG	317.5	361.0	0.07	0.00	0.07	1.51E-06	1.57E-06
tan	X	9113462	C	481.0	488.0	0.03	0.09	0.06	3.34E-05	

Supplementary Table 4. Differences in indel frequencies between light and dark phenotypes for indels with $p < 0.0001$. Indels were called by remapping the regions near the tan, bab and ebony loci (within 20kb of the middle of the coding sequence of these genes) with Dindel. As Dindel divides the reads in these regions into windows, some indels occur in more than one window. In these cases, the presence and abscence counts of these indels were usually quite similar, and so we give the average coverage and frequency and the minimum and maximum p-values. Similarly, indels with adjacent positions are also merged into a single entry, and the same information is reported for these.
