# Supplementary material for: A Genome-Wide, Fine-Scale Map of Natural Pigmentation Variation in Drosophila melanogaster
Source: PLoS Genet. 2013 Jun 6;9(6):e1003534. doi: 10.1371/journal.pgen.1003534 (PMC3674992; doi:10.1371/journal.pgen.1003534)
Supplement: Table S5 — Transposable element insertion frequencies in the pigmentation genes tan, bab1 and ebony. These regions are defined, as before, as 20 kb up- and down- stream of the center of the coding sequence of each gene. Transposable element insertions found in these regions using the method described in Text S1, except that insertions detected in only one replicate are not shown. A CMH test was performed on the presence and absence counts for each detected insertion. Some frequency estimates may be unreliable, as they come from insertions that overlap with other insertions or, in one case, from an INE-1 insertion, which are typically very short; these are nevertheless shown for completeness. (PDF) [file pgen.1003534.s017.pdf]

Gene	Chrom	Estimated position	Transposable element family	Flybase ID	Population	Total light coverage	Total dark coverage	Presence frequency light	Presence frequency dark	Frequency difference	CMH p-value	
bab	3L	1090285.75	FR	mdg1	both	498	305	0.01	0.03	0.018	3.7E-04	
bab	3L	1083542.5	FR	opus	Bolzano	142	125	0.00	0.04	0.040	0.022	*
bab	3L	1098821	FR	p-element	Bolzano	264	264	0.00	0.03	0.027	0.026	
bab	3L	1087483	FR	I-element	Bolzano	255	254	0.00	0.02	0.024	0.040	
bab	3L	1086188	FR	roo	Bolzano	68.5	79.5	0.02	0.07	0.047	0.358	*
bab	3L	1083087	R	roo	Bolzano	131	148	0.00	0.01	0.007	0.398	
bab	3L	1080939	FR	412	Vienna	179	56	0.02	0.05	0.037	0.406	
bab	3L	1083501	R	F-element	Bolzano	83	53	0.02	0.06	0.033	0.480	*
bab	3L	1086290.5	FR	Tabor	Bolzano	68	86	0.00	0.02	0.023	0.598	*
bab	3L	1098887.75	RFR	jockey	Vienna	90	255	0.02	0.03	0.009	0.797	
bab	3L	1069502.5	FR	412	Bolzano	240	233	0.02	0.02	0.001	0.861	
bab	3L	1078942	R	Doc	Bolzano	86	94	0.02	0.01	0.013	0.876	
bab	3L	1086505	F	Cr1a	Bolzano	111	116	0.00	0.01	0.009	0.946	
bab	3L	1076828	F	Ivk	Bolzano	130	140	0.02	0.01	0.008	0.971	
bab	3L	1102390	F	p-element	Bolzano	107	114	0.01	0.02	0.008	0.977	
ebony	3R	17080912.8	FRFRFR	INE-1	both	460	444	0.19	0.84	0.655	2.2E-13	*
ebony	3R	17082141.8	FRF	Burdock	both	273	275	0.03	0.00	0.033	0.009	*
ebony	3R	17082059	R	roo	Bolzano	148	159	0.03	0.00	0.034	0.050	*
ebony	3R	17082115.5	FR	Doc	Bolzano	177	216	0.01	0.04	0.031	0.083	*
ebony	3R	17059246	FR	I-element	Bolzano	199	286	0.00	0.02	0.021	0.133	
ebony	3R	17082065	F	hobo	Bolzano	19	17	0.16	0.00	0.158	0.219	*
ebony	3R	17049287	F	S-element	Bolzano	130	132	0.02	0.00	0.023	0.230	
ebony	3R	17076452	R	roo	Bolzano	69	67	0.04	0.00	0.043	0.356	
ebony	3R	17064359	FR	Max-element	Bolzano	254	270	0.02	0.01	0.013	0.484	
ebony	3R	17082055	R	hopper	Bolzano	117	140	0.03	0.01	0.020	0.487	*
ebony	3R	17074652	F	roo	Bolzano	23	26	0.13	0.04	0.092	0.701	
ebony	3R	17082321.3	FRF	jockey	Vienna	158	117	0.03	0.03	0.000	0.967	
ebony	3R	17064810.3	RRR	mdg1	both	214	251	0.02	0.02	0.003	1.000	
tan	X	9113715	R	roo	Bolzano	57	89	0.11	0.00	0.105	0.007	
tan	X	9112000	R	F-element	Bolzano	115	124	0.04		0.043	0.189	
tan	X	9106434	FR	flea	Bolzano	173	168	0.01	0.03	0.024	0.194	*
tan	X	9108802	FR	I-element	Vienna	118	52	0.02	0.06	0.041	0.333	
tan	X	9106374	F	Transpac	Bolzano	8	6	0.38	0.00	0.375	0.386	*
tan	X	9107652.5	RF	jockey	Bolzano	138	132.5	0.02	0.00	0.018	0.480	*
tan	X	9107662.5	FR	roo	Bolzano	161	185	0.02	0.03	0.014	0.543	*
tan	X	9111965	RF	opus	Bolzano	114.5	110	0.02	0.01	0.008	0.597	*
tan	X	9111730	R	Idefix	Bolzano	17	19	0.12	0.16	0.040	0.770	*
tan	X	9111867.25	RFR	297	both	58	76.5	0.02	0.07	0.055	0.976	*
tan	X	9107632	FR	hobo	Bolzano	163	210	0.02	0.02	0.005	0.990	*

Supplementary Table S5. Transposable element insertion frequencies in the pigmentation genes tan, bab and ebony. These regions are defined, as before, as 20kb up- and down- stream of the center of the coding sequence of each gene. Transposable element insertions found in these regions using the method described in the text are shown, except that insertions detected in only one replicate are not shown. A CMH test was performed on the presence and absence counts for each detected insertion. Some frequency estimates may be unreliable, as they come from insertions that overlap with other insertions or, in one case, from an INE-1 insertion, which are typically very short; these are nevertheless shown for completness.
