# Supplementary material for: A Genome-Wide, Fine-Scale Map of Natural Pigmentation Variation in Drosophila melanogaster
Source: PLoS Genet. 2013 Jun 6;9(6):e1003534. doi: 10.1371/journal.pgen.1003534 (PMC3674992; doi:10.1371/journal.pgen.1003534)
Supplement: Table S6 — Estimated effects of SNPs with FDR<0.05. The effect of each SNP is ranked by its log odds ratio, as discussed in the text. The p-values and odds ratios come from the CMH test used for the main analysis, and ranks are based on the magnitude of the log of the odds ratio. We use the magnitude of the log of the odds ratio to estimate the rank of the effect, as is standard, for the following reason: For a SNP of no effect, the odds ratio should be 1 (and the log odds ratio equal to 0). The size of the estimated effect increases as the odds ratio deviates in either direction from 1 (and the log odds from 0). But, as odds ratios are restricted between 0 and infinity, this deviation is a non-linear function of the size of the real effect. The log odds, in contrast, is symmetric about 0, and the size of this deviation in either direction is expected to be proportional to the size of the effect. Though the effects here are systematically overestimated, the rankings appear to be robust as discussed in Text S2. (PDF) [file pgen.1003534.s018.pdf]

chr	pos	gene	CMH pvalue	pooled odds ratio	|log odds ratio|	rank
X	9121094	*tan*	2.06E-56	0.032061469	3.440100309	1
X	9121129	*tan*	1.39E-58	0.039765166	3.224763976	2
X	9120922	*tan*	6.84E-51	0.082052445	2.500396661	3
X	9121338	*tan*	3.63E-34	0.122945162	2.09601686	4
X	9121177	*tan*	1.20E-34	0.148691752	1.905879895	5
X	9121191	*tan*	9.82E-34	0.151992754	1.88392243	6
3L	1085454	*bab*	7.63E-23	6.209605029	1.826097292	7
X	9117751	*tan*	2.11E-21	0.184280876	1.691294184	8
X	9118408	*tan*	9.94E-22	0.221862947	1.505695442	9
X	9119116	*tan*	5.00E-23	0.223879694	1.496646451	10
X	9120204	*tan*	1.10E-23	0.226983719	1.482876985	11
X	9119071	*tan*	5.42E-29	0.231795536	1.461899608	12
X	9119157	*tan*	5.56E-23	0.238518146	1.433309889	13
X	9119160	*tan*	4.46E-23	0.240569347	1.424746885	14
X	9121584	*tan*	5.19E-23	0.253920075	1.370735728	15
X	9120683	*tan*	2.78E-21	0.288172139	1.244197273	16
X	9120730	*tan*	1.97E-21	0.301263434	1.199770201	17
