# Supplementary material for: A Genome-Wide, Fine-Scale Map of Natural Pigmentation Variation in Drosophila melanogaster
Source: PLoS Genet. 2013 Jun 6;9(6):e1003534. doi: 10.1371/journal.pgen.1003534 (PMC3674992; doi:10.1371/journal.pgen.1003534)
Supplement: Text S1 — Supplementary methods and results. Additional detail on the sequencing method, removal of D. simulans contamination, statistical analysis for association mapping and FDR correction. Also includes detailed methods on the indel and TE insertion analysis and results. (PDF) [file pgen.1003534.s019.pdf]

Text S1. Supplementary methods for sequence analysis, FDR correction, indel and TE insertion analysis.

DNA extraction and sequencing, detailed methods

For extraction of genomic DNA, each pool of female flies was homogenized with an Ultraturrax T10 homogenizer (IKA-Werke, Staufen, Germany) in a high salt extraction buffer [1]. After RNase A treatment (Qiagen, Hilden, Germany) the DNA was purified by chloroform extraction and Ethanol precipitation. For each sample, 5 µg of genomic DNA were sheared with a Covaris S2 device (Covaris, Inc. Woburn, MA). Paired-end libraries were prepared using the Paired-End DNA Sample Preparation Kit (Illumina, San Diego, CA; Vienna sample) or the NEBNext DNA Sample Prep modules (New England Biolabs, Ipswich, MA; Bolzano samples) following the manufacturers´ instructions. After end repair, 3´-adenylation and ligation of Illumina paired-end adapters ligation products of ~350 bp were gel-purified using the Qiagen gel extraction kit (Qiagen; Hilden, Germany). To avoid guanine-cytosine (GC) bias the gel slice was dissolved at room temperature instead of heating. Libraries were amplified using Phusion DNA polymerase (New England Biolabs, Ipswich, MA) and protocol: polymerase activation (98°C for 30 s), followed by 10 cycles (denaturation at 98°C for 10 s, annealing at 65°C for 30 s, and extension at 72°C for 50 s) with a final, 7 min. extension at 72°C. Libraries were purified and quantified using the Qubit HS Assay Kit (Invitrogen, Carlsbad, CA, USA).

All samples were prepared as described above except that a ligation product of ~450 bp was purified for the light, dark and control samples from replicate I of the Viennese sample, and custom-modified barcoded paired-end

adapters were used for the light, dark and control samples from replicate I of the Bolzano sample (sequence and protocol are available upon request). Libraries were sequenced using the 2x101 bp or 2x151 bp paired-end protocol on either a Genome Analyzer IIx or a HiSeq2000.

Removal of *D. simulans* contamination

As described in the main text, we filtered for *D. simulans* contamination by competitively remapping reads to five *D. melanogaster* and five *D. simulans* genomes using GSNAP [2,3]. As *D. simulans* is lighter overall for abdominal pigmentation than *D. melanogaster* [4], contamination can lead to an enrichment of *D. simulans* reads in the light samples, and potentially to false-positive SNPs between light and dark samples. In detail, we converted the sorted bam output from the BWA mapping to fastq format using bam2fastq v1.1.0 (http://www.hudsonalpha.org/gsl/software/bam2fastq.php), including only reads with mapping qualities of 20 or higher, and which mapped in proper pairs. These reads were remapped to five *D. melanogaster* genomes (including the reference genome v.5.18 cited above and those of the MW6, MW28, RAL360, RAL732 strains from the *Drosophila* Population Genomics Project (http://www.dpgp.org/) and five *D. simulans* genomes (unpublished data), using GSNAP version 2011-12-28 [2,3]. Reads were identified as *D. melanogaster* if they mapped to at least one of five *D. melanogaster* genomes and not to any one of five *D. simulans* genomes. Reads mapping to at least one genome of each species but displaying the same or a higher mapping quality in the alignment to *D. melanogaster* were also considered to be of *D. melanogaster* origin. The reads identified as *D. melanogaster* were then extracted from the original sorted bam files for further processing.

To evaluate the effectiveness of this filtering procedure, we estimated the extent of *D. simulans* contamination before and after filtering (Figure S11). This evaluation was based on more than 11,000 sites with fixed differences between *D. melanogaster* and *D. simulans*, obtained from three *D. melanogaster* and three *D. simulans* populations (unpublished results). The fixed differences were identified by mapping these population samples against the *D. melanogaster* reference genome and considering only sites where the nucleotide state was different between the species, but monomorphic within species (except that we included sites with minor alleles occurring at most twice). Importantly, these species-specific sites were obtained from sources other than the 10 genomes used for filtering the reads.

Coverage of each replicate

The average coverage of the different replicates, after undergoing all filtering procedures, was approximately Vienna replicate 1, light: 90.49 and dark: 70.33, replicate 2, light: 102.99, and dark: 99.56; Bolzano replicate 1, light: 41.23 and dark: 30.30, replicate 2, light: 139.21, and dark: 157.60; replicate 3, light: 138.77, and dark: 151.67. For the reference populations, the average coverage was in Vienna replicate 1: 120.67, replicate 2: 165.54 and replicate 3: 126.05; Bolzano replicate 1: 54.13, replicate 2: 141.63 and replicate 3: 162.94.

Association mapping and FDR correction

We tested SNPs showing an association with pigmentation using the Cochran-Mantel-Haenszel (CMH) test, as described in the main text. To test the populations individually, we again used the CMH test to assess significance across the two replicates from Vienna and the three from Bolzano. The results of

these analyses were essentially similar whether the filtering criteria used were applied to the individual populations, or instead included all the SNPs that survived the filtering criteria for the joint analysis, as long as they remained polymorphic in the single samples. For the FDR correction for single populations, we used the SNPs that survived filtering criteria applied to the single populations.

To correct for multiple tests, we estimate the rate of false-discoveries for a given p-value, i.e., the FDR. To obtain the FDR, we must first estimate the distribution of the p-values from the CMH test under the null hypothesis. Under the null, there are two sources of sampling variation. The first source is the sampling of alleles between phenotypes; under the null model, the n copies of an allele assigned to the light or dark sample will be binomially distributed with parameters n and p, with $p = P($pool | allele$)$, the conditional probability of assigning an allele to the light or dark pools. We can therefore model this source of variation with a simple binomial model.

The second source of sampling variation occurs if there is a bias for or against sampling an allele in one of the pools, as might happen if an allele is over- or under-represented in a pool due to population structure or technical issues (e.g., over- or under- amplification of an allele during the PCR step). That is, unlike in the simple binomial model, there might be random background variation in $P($pool | allele$)$. We can account for this second source of sampling variation by expanding the binomial model above, using a beta-binomial model. In this approach, the $P($pool | allele$)$ has the same mean value as under the simple binomial model, but a non-zero variance. As suggested by the name of the beta-binomial model, the variation in $P($pool | allele) is modeled by drawing

values for this parameter from a beta distribution, a flexible two parameter distribution (parameters α and β), with the mean equal to $\alpha/(\alpha+\beta)$, and a variance that decreases with increasing α and β [equal to $(\alpha \beta)/\{(\alpha+\beta)^2(\alpha+\beta+1)\}$]. In the case where α and β become very large, the variance tends toward 0, and the beta-binomial model collapses to the simple binomial model.

Here, where the coverages are very close to equal between the light and dark pools, the mean of *P(*pool | allele*)* should be 0.5 under the null, so we use a binomial with $\alpha = \beta$, giving a mean for the beta of 0.5 and a variance of $1/(8\alpha + 4)$. (Note, however, that the ratio of these two parameters can be adjusted to account for unequal coverages). *A priori*, we don't know what variance to use, but we can change this α to optimize the fit of the beta-binomial model to the real data.

To test different values of α, and ultimately to perform the FDR correction, we therefore simulate data sets according to the beta-binomial model, allowing us to obtain a distribution of *p*-values under the null hypothesis that we can compare to the observed distribution of *p*-values. Each data set was simulated using a single value of α for the whole data set, α_{null} , and each simulated data set contains the same number of SNPs as in the real data (~3.3 million), with each SNP represented by random 2 x 2 x 5 tables (as there are 2 phenotypes, 2 alleles, and 5 replicates). The coverages and allele frequencies for each SNP are taken from the data, as described below (i.e., the column sums and overall table sums of the simulated tables correspond to those from the data).

For each SNP, the random tables were generated using the following procedure:

(i) For a single replicate, we generated a random 2 x 2 table, with the two nominal variables being the phenotype (light *vs.* dark) and allelic state (A1 *vs*. A2).

(ii) To obtain the 2 values in the table for the A1 allele, we need to distribute the n_{A1} copies of this allele randomly over the light and dark phenotypes (with n_{A1} obtained from the data). We assign k of the n_{A1} copies to the dark pool by drawing a random k from a binomial distribution. This binomial has parameters $n=n_{A1}$ and $p=P($dark | A1$)$. We obtain $P($dark | A1$)$ by drawing it from a beta distribution, using α_{null} as the value for both parameters. We then assign the remaining $n_{A1}-k$ copies of A1 to the light pool.

(iii) To fill out the remainder of the 2 x 2 table, we repeat this procedure for the second allele, A2, drawing a new value from the same beta distribution to represent $P($dark | A2$)$.

We repeat this procedure for each of the 5 replicates in the real data, and then performed a CMH test on the resulting 2x2x5 tables, yielding a *p*-value. The null distribution evaluated for each α parameter consists of *p*-values generated for all ~3.3 million SNPs in this way.

To select an appropriate α value to use for the FDR correction, we generated these null-distributions under a range of different values for α. We evaluated the fit of these null distributions to the real data by taking the chi-square distance between the null and observed *p*-value distributions, using counts obtained from the *hist* function in R (http://www.r-project.org), with 1000 identical bins used for all comparisons. Examining the fit of the whole range of *p*-values yields a conservative FDR cutoff, as is reflected in the quantile-quantile plot (Figure S12).

Finally, we estimate the FDR for the most significant SNPs. To do this, we generated the equivalent of 10 data sets under the null using the best-fit alpha value. Simulation of multiple data sets is standard when the FDR is obtained by simulation [5], p. 689), as the FDR is estimated using the values in the extreme tails of the null distribution. Since these samples are rare, simulating multiple data sets helps ensure that enough tail samples are obtained for an accurate FDR correction. The FDR for the ith ranked SNP was calculated by obtaining the number of simulated SNPs with lower *p*-values than this SNP, and adjusting this number to reflect the average number obtained in sample of the same size as the real data set (i.e., dividing by 10 in this case). This represents the average number of SNPs from the null distribution that are expected to have a value at least as extreme as that of the real SNP. This average is then divided by the rank of the SNP to obtain the FDR (e.g., if, for the *p*-value corresponding to the SNP with rank 10 from the data, an average of 0.5 simulated *p*-values are equal to it or smaller, the FDR for that SNP is 0.5/10, or 0.05). The entire process—selecting an appropriate alpha value, generating a null-distribution of *p*-values, and calculating FDRs—was repeated for the two population samples independently and for each individual replicate, except that for individual replicates a Fisher's Exact Test was used in place of the CMH test.

We further tested for associations between pigmentation and indels or transposable element (TE) insertions, but limited these tests to the *tan*, *bab*, and *ebony* regions. The rationale for this limitation was that, if an indel or TE was strongly associated with pigmentation, at least some nearby SNPs would also show associations, and all of the significant and almost all of the highly ranked SNPs were confined to this region. Further, these analysis required careful hand

curation, necessarily restricting their scope. Finally, we did not consider replicate I from Bolzano, as indel and TE insertion detection are more sensitive to coverage than SNP detection, and as this replicate had much lower coverage than the others.

The presence of indels was investigated using Dindel v1.01 [6]. We obtained library insert size distributions from the bam files of each sample separately, and indels were identified from the merged bam files (using the do-pooled option designed for pools of individuals). Bam files from each sample treated separately were then realigned to the indels previously identified in the merged data. We converted the output file into a synchronized file and performed the CMH test on the two replicates from Vienna and the replicates II and III from Italy simultaneously (Table S4).

TE insertion frequencies were assessed separately in Vienna and in Bolzano using PoPoolation TE [7]. Briefly, paired-end reads of each sample were mapped with bwa sw and a sam file was created with the script samro.pl. The forward and reverse insertions were identified using the script identify-te-insertsites.pl and crosslinked with crosslink-te-sites.pl. For this last script, a minimum distance of 100 and a maximum distance of 300 was used for the two replicates from Italy (as their insert size is the same between the two replicates), a minimum distance of 75 and a maximum distance of 450 for Vienna RI and a minimum distance of 75 and a maximum distance of 350 for Vienna RII (as the insert sizes of those last two replicates differ). The script estimate-polymorphism.pl allowed the estimation of frequencies in each sample separately (using a minimum mapping quality of 15).

Each sample had to be treated separately for the TE identification, as the method does not allow for differences in insert size. We therefore manually inspected the TE insertions from each sample, and merged all TE insertions from the same family with estimated positions that fell within 300 bp of each other. We then performed a CMH test on all insertions that occurred in at least two replicates (Table S5).

References

1. Miller SA, Dykes DD, Polesky HF (1988) A simple salting out procedure for extracting DNA from human nucleated cells. Nucleic Acids Res 16: 1215.

2. Wu TD, Watanabe CK (2005) GMAP: a genomic mapping and alignment program for mRNA and EST sequences. Bioinformatics 21: 1859-1875.

3. Wu TD, Nacu S (2010) Fast and SNP-tolerant detection of complex variants and splicing in short reads. Bioinformatics 26: 873-881.

4. Gibert P, Moreteau B, Moreteau JC, David JR (1996) Growth temperature and adult pigmentation in two *Drosophila* sibling species: An adaptive convergence of reaction norms in sympatric populations? *Evolution* 50: 2346-2353.

5. Hastie R, Tibshirani R, Friedman J (2009) The Elements of Statistical Learning Bickel P, Diggle P, Fienberg S, Gather U, Olkin I et al., editors. New York, NY: Springer-Verlag.

6. Albers CA, Lunter G, MacArthur DG, McVean G, Ouwehand WH, et al. Dindel: accurate indel calls from short-read data. Genome Res 21: 961-973.

7. Kofler R, Betancourt AJ, Schlötterer C (2012) Sequencing of pooled DNA samples (Pool-Seq) uncovers complex dynamics of transposable element insertions in *Drosophila melanogaster*. PLoS Genet 8: e1002487.
