# Supplementary material for: A Genome-Wide, Fine-Scale Map of Natural Pigmentation Variation in Drosophila melanogaster
Source: PLoS Genet. 2013 Jun 6;9(6):e1003534. doi: 10.1371/journal.pgen.1003534 (PMC3674992; doi:10.1371/journal.pgen.1003534)
Supplement: Text S2 — Description of highest ranked SNPs. Describes the characteristics, allele frequencies before selection, and the estimation of effects for the highly ranked SNPs. (PDF) [file pgen.1003534.s020.pdf]

Text S2. Description of highest ranked SNPs

Characteristics of highly ranked SNPs

For the top 100 ranked SNPs, we provide tables showing the rank, the location (chromosome and position), the reference and alternative nucleotide, the gene(s) in which they lie (gene ID and gene name), the effect on the gene (synonymous coding, intron, etc.), the change in amino acid and codon (if in coding sequence) and the *p*-value from our analysis. The colors indicate which, if any, pigmentation gene the SNP is near (blue: *tan*; red: *bab1*; yellow: *ebony*; no color: none). These values are given for the analysis of the combined samples (Table S1), and for the analyses of the Viennese (Table S2) and Bolzano (Table S3) samples.

Frequencies of highly ranked SNPs before selection, estimated from control samples

We also examined the frequencies of these SNPs in control samples from the Vienna and Bolzano populations. The minor allele frequencies (MAF) of the 17 significant SNPs range from 7.8 to 50.0% in the Viennese populations, and from 11.0 to 46.0% in the Bolzano populations. We expect to have the most power to detect real associations when the MAF is close to 50. Consistent with this idea, the MAF of highly ranked and significant SNPs in the control populations are skewed toward high MAFs compared to a random subset of SNPs (Figure S10).

Estimation of effects for significant SNPs

Typically, when using contrasts between groups in GWAS, effects are estimated using the odds ratio in a logistic regression. Similarly, we can obtain pooled odds ratio estimates from the CMH test for the significant SNPs (Table S6); however, there are several reasons to be cautious in interpreting these estimates. The primary issue is due to the pooling; because the total genotypes of the individuals at all SNPs in the light and dark pools are not known, we can not estimate the

effect of a single SNP independently while controlling for genotypes at other SNPs in the genetic background (as is done statistically in logistic regression). If we could rely on the genetic background being randomized between the pools, we might expect the pooled odds ratio estimates to be unbiased: e.g., a study comparing effects of alleles associated with breast cancer from individual data (estimated with logistic regression) and pooled data (estimated with an odds ratio from a single homogeneity table) shows that the estimates from the pooled data, though noisier, are similar to those from logistic regression [1]. However, as we have selected for light and dark individuals in these pools, we likely cannot rely on a randomized genetic background, and it is probably the case that the dark individuals have more dark alleles in the genetic background. Moreover, some nearby SNPs likely appear on the same haplotypes, particularly at tan, so some of these estimates likely reflect the same underlying effects.

To investigate sources of bias in the odds ratio estimates of the CMH test, we performed some simple simulations resembling our experiment (Figure S4). Comparing the two kinds of estimates shows that those from the CMH test are both usually higher and also noisier than those from the estimates from logistic regression. Thus, the magnitude of the estimates can not be taken too seriously. Nevertheless, the rank order of the estimates is in general the same as the rank order of effects of the SNPs, and the correspondence is not much worse than that of the estimates from logistic regression [Spearman's rank correlations on the 500 simulated replicates from Figure S4 between the true effect of the SNP and the estimated effect show that, for the logistic regression estimates: mean r_S = 0.931 (range from 0.792 to 0.990), for the CMH estimates: mean r_S = 0.885 (range from 0.767 to 0.967)]. While not a very stringent requirement, this correlation of ranks is probably the most relevant aspect of these estimates. Unlike estimating the risk of developing a genetic disease, estimating the risk of a fly being dark or light enough to fall into one of our extreme pools has almost no biological meaning outside this

experiment. But, once the SNPs have been detected, the ranks of effects provide some basis for deciding which SNPs represent the most promising candidates for follow-up studies.

References

1. Huang Y, Hinds DA, Qi L, Prentice RL (2010) Pooled versus individual genotyping in a breast cancer genome-wide association study. Genet Epidemiol 34: 603-612.
