# Supplementary material for: A Genome-Wide, Fine-Scale Map of Natural Pigmentation Variation in Drosophila melanogaster
Source: PLoS Genet. 2013 Jun 6;9(6):e1003534. doi: 10.1371/journal.pgen.1003534 (PMC3674992; doi:10.1371/journal.pgen.1003534)
Supplement: Text S3 — Results of separate analysis of Bolzano and Vienna populations. Describes additional results obtained for association mapping of pigmentation SNPs done separately for the Bolzano and Vienna samples. (PDF) [file pgen.1003534.s021.pdf]

Text S3. Results of separate analysis of Bolzano and Vienna populations

In addition to the main analysis, which included both the Bolzano and Vienna populations, we also examined these samples separately. For this analysis, we first filtered SNPs based on the combined data (e.g., minor allele frequency cutoffs, indel masking, etc.), and then re-extracted those SNPs from the individual populations. As a result, analysis of the two individual populations included the same SNPs as the combined analysis (though not all SNPs were polymorphic in both populations). This procedure resulted in the analysis of 2,886,649 SNPs in the Vienna population and 3,103,730 SNPs in the Bolzano population. We applied the CMH test as described in the main text for the combined data (Figures S1 and S2).

In the combined analysis, all else being equal, we have more power to detect significant associations when SNPs are associated with pigmentation in both populations *vs.* only one of the populations. The combined analysis, then, will tend to obscure associations at any SNPs that differ in their associations between the populations. But, by analyzing the populations separately, we can attempt to recover evidence for population specific associations. For this analysis, we examine the ranks of the *p*-values within a population, rather than the *p*-values themselves, as there are differences in power between the populations. Further, to be conservative, we limit this analysis to the three regions where we have good reason to suspect false-negative associations, i.e., the *tan*, *bab* and *ebony* regions. We consider SNPs to have population specific associations, then, if they are highly ranked (among the top 100) in either Bolzano or Vienna samples, but not in the other sample.

As Figure S3 shows, there were several SNPs with population-specific associations in all three of the pigmentation genes, particularly in the promoter region of *bab* (Figure S3B, right side), and in *ebony* (Figure S3C). No SNP in these two promoter regions was below the FDR cutoff of 0.05 in any of the three analyses (in Bolzano and Vienna analyzed separately, or in the combined analysis). The lack of significance in the single population analyses is likely attributable to a lack of power (as a cluster of SNPs near pigmentation genes would be unexpected otherwise). The lack of significance in the joint analysis, however, may be due to the population-specificity of the associations.

The biological nature of such associations is unlikely to be that two European *D. melanogaster* populations use entirely different genetic pathways for female abdominal pigmentation. Nevertheless, any of several more subtle differences in genetic architecture might lead to population-specific associations. For example, there may be differences in allele frequencies, either at the SNP sites themselves, or in an epistatic locus in the genetic background. In either case, the result would be that some SNPs might have a large effect in only one population. The first explanation of an epistatic locus with large frequency differences between the populations cannot be examined, but appears rather unlikely given the overall similarity of cosmopolitan *D. melanogaster* populations [1] including the two populations of this study (not shown). The second explanation is that there are differences in allele frequencies at SNPs with population-specific effects, but allele frequencies in the unselected controls from the same populations were similar (not shown). An alternative explanation is that the SNPs are linked to a causal variant, but that the causal variant or variants appear on different haplotypes in the two populations. Finally, the

population-specific SNPs may themselves cause pigmentation differences, as part of multiple *cis*-acting modules occurring on different haplotypes in the two populations.

References

1. Orozco-terWengel P, Corander J, Schlötterer C (2011) Genealogical lineage sorting leads to significant, but incorrect Bayesian multilocus inference of population structure. Mol Ecol 20: 1108-1121.
