# Supplementary material for: A Genome-Wide, Fine-Scale Map of Natural Pigmentation Variation in Drosophila melanogaster
Source: PLoS Genet. 2013 Jun 6;9(6):e1003534. doi: 10.1371/journal.pgen.1003534 (PMC3674992; doi:10.1371/journal.pgen.1003534)
Supplement: Text S4 — Simulation methods. Describes detailed methods for the simulations for examining the effects of different initial allele frequencies, and different genetic architectures of the trait (including the number of loci involved, the distribution of their effects, and different contributions of the environment to the trait). (PDF) [file pgen.1003534.s022.pdf]

Text S4. Simulation methods and results

To investigate factors affecting the power to detect associations and the repeatability of our results, we performed simulations mimicking the experiment, but varying five factors: the experimental design (sequencing extreme phenotypes *vs.* sequencing one extreme and a control sample), the penetrance of the trait (with p = 0.1, 0.5, or 1), the number of loci affecting the trait (l = 10, 20, or 100), the distribution of phenotypic effects of the loci (either equal effects or exponential), and the frequencies of the causal alleles [which either had unrestricted initial frequencies before selection (mf = 0), or were required to be at least moderately common (mf = 0.2)].

To perform these simulations, we first simulated a sample representing 8000 *Drosophila melanogaster* genomes using fastsimcoal v. 1.1.2 [1], with recombination rates and diversity specified in 100kb windows. Recombination rates were obtained from the *Drosophila* recombination rate calculator v. 2.2 [2]. Diversity levels were specified by changing the mutation rate parameter according to the average pairwise diversity in a window of the control sample (*i.e,* the sample not selected for pigmentation) from Vienna, as calculated using the Variance-sliding.pl from PoPoolation1 [3], in 100kb non-overlapping windows, with the minimum count set to 2 and a correction applied for exclusion of low frequency alleles. To avoid fluctuations in diversity due to poorly covered regions, we fit linear models to recombination rate and diversity for each chromosome arm ($p < 1e^{-10}$ in all cases), and use the predicted diversity values in the simulations. Two such samples of 8000 genomes were simulated, with each used for half of the simulations, to avoid dependence on a single sample. We then

used a custom python script to assign causal loci and phenotypes to individuals, and to select individuals with extreme phenotypes. Individual phenotypes were calculated with the genotypic contribution to the phenotypic trait value consisting of the sum of the effects at each causal locus, and the environmental component of the trait determined by standard random normal deviate.

Results from the simulations are shown in Figures S5-S7, summarized as receiver operator curves (ROC). Note that the simulations are different from the actual experiment in several ways. Specifically, the simulated individuals are haploid, and the sequence coverage is uniform genome-wide. Nevertheless, contrasts between the different simulated cases provide some guidance for when our approach is more or less powerful. In general, the results show that significant SNPs are enriched for causative SNPs in the simulations (i.e, the ROC curves are always above the red line in the plot, which corresponds to equal false and true positive rates). The proportion of causative SNPs detected increases when they are required to have common alleles (at a frequency of between 20 and 80%), when the causative alleles have high penetrance, and when the trait has a simple genetic basis (compare ROC curves where 10 or 20 causative sites underlay the trait *vs.* when 100 causative sites do). Including SNPs within 100 base pairs of the causative SNPs amongst the true positives (as these would act as markers for nearby causative variants) appears to only slightly increase the power of the method (not shown). In general, excluding low recombination sites from the analysis greatly improves both the false and true positive rates (compare the solid and dotted lines in the figures), likely due to haplotype structure in those regions making distinctions between true and false positives difficult. Note that *(i)* the simulations do not include gene conversion, so that

linkage disequilibrium in these regions is much stronger than is likely to be true for the real data, and *(ii)* none of our significant SNPs from the real analysis fell into low recombination regions. Finally, note that selecting two extreme phenotypes (the black lines in the figures) generally results in more power than comparing a selected to a control sample, though it may also slightly increase the false positive rate.

We also performed simulations to investigate the repeatability of our results when the experiment is performed repeatedly. Simulations were performed as above, except that each of the simulations was repeated 20 times, with a fixed random seed such that the causal loci and their effects were identical among replicates. Some stochastic effects remain: the division of flies into replicates and change in individual phenotypes due to the environment varied between simulations (the proportion of the phenotype due to the environment was set to 0.5). As the strongest candidates from the analysis of the data are at intermediate frequencies in the unselected reference populations, we required the causal loci to have an MAF between 0.2 and 0.5. We performed 12 runs (of 20 replicates each) for each of the cases shown in Figures S8 and S9, asking if the SNPs with true effects are consistent among the 20 replicates. The consistency of results between runs was summarized as the range of p-values from the CMH test among the 20 replicates of each run. These results show, not surprisingly, that repeatability is affected by: *(i)* the number of SNPs with true effect, *(ii)* the initial allele frequencies and, *(iii)* when SNPs have different effects, the magnitude of the effect on the phenotype. Both higher initial frequency and stronger effects increase the repeatability of the experiment; larger numbers of SNPs affecting the trait reduce it. In general, SNPs that show high significance in

at least one replicate tend to be highly repeatable (the black points in the plots show a small range of *p*-values) and those with high minimum *p*-values tend to be reliably non-significant.

References

1. Excoffier L, Foll M (2011) fastsimcoal: a continuous-time coalescent simulator of genomic diversity under arbitrarily complex evolutionary scenarios. Bioinformatics 27: 1332-1334.
2. Fiston-Lavier AS, Singh ND, Lipatov M, Petrov DA (2010) *Drosophila melanogaster* recombination rate calculator. Gene 463: 18-20.
3. Kofler R, Orozco-terWengel P, De Maio N, Pandey RV, Nolte V, et al. (2011) PoPoolation: a toolbox for population genetic analysis of next generation sequencing data from pooled individuals. PLoS One 6: e15925.
